# Multi-sequence generative adversarial network: better generation for enhanced magnetic resonance imaging images

**DOI:** 10.3389/fncom.2024.1365238

**Published:** 2024-05-22

**Authors:** Leizi Li, Jingchun Yu, Yijin Li, Jinbo Wei, Ruifang Fan, Dieen Wu, Yufeng Ye

**Affiliations:** ^1^South China Normal University-Panyu Central Hospital Joint Laboratory of Basic and Translational Medical Research, Guangzhou Panyu Central Hospital, Guangzhou, China; ^2^Guangzhou Key Laboratory of Subtropical Biodiversity and Biomonitoring and Guangdong Provincial Engineering Technology Research Center for Drug and Food Biological Resources Processing and Comprehensive Utilization, School of Life Sciences, South China Normal University, Guangzhou, China; ^3^Medical Imaging Institute of Panyu, Guangzhou, China

**Keywords:** generative adversarial network, magnetic resonance imaging, multimodal, convolutional neural network, contrast-enhanced magnetic resonance sequence

## Abstract

**Introduction:**

MRI is one of the commonly used diagnostic methods in clinical practice, especially in brain diseases. There are many sequences in MRI, but T1CE images can only be obtained by using contrast agents. Many patients (such as cancer patients) must undergo alignment of multiple MRI sequences for diagnosis, especially the contrast-enhanced magnetic resonance sequence. However, some patients such as pregnant women, children, etc. find it difficult to use contrast agents to obtain enhanced sequences, and contrast agents have many adverse reactions, which can pose a significant risk. With the continuous development of deep learning, the emergence of generative adversarial networks makes it possible to extract features from one type of image to generate another type of image.

**Methods:**

We propose a generative adversarial network model with multimodal inputs and end-to-end decoding based on the pix2pix model. For the pix2pix model, we used four evaluation metrics: NMSE, RMSE, SSIM, and PNSR to assess the effectiveness of our generated model.

**Results:**

Through statistical analysis, we compared our proposed new model with pix2pix and found significant differences between the two. Our model outperformed pix2pix, with higher SSIM and PNSR, lower NMSE and RMSE. We also found that the input of T1W images and T2W images had better effects than other combinations, providing new ideas for subsequent work on generating magnetic resonance enhancement sequence images. By using our model, it is possible to generate magnetic resonance enhanced sequence images based on magnetic resonance non-enhanced sequence images.

**Discussion:**

This has significant implications as it can greatly reduce the use of contrast agents to protect populations such as pregnant women and children who are contraindicated for contrast agents. Additionally, contrast agents are relatively expensive, and this generation method may bring about substantial economic benefits.

## 1 Introduction

Magnetic resonance imaging (MRI) is one of the most used imaging protocols in medical diagnosis, especially used in the brain disease. It is a multifunctional imaging technique that can generate different tissue contrast based on acquisition parameters (e.g., T1-weighted, T2-weighted, Fluid Attenuated Inversion Recovery and T1-weighted contrast enhance). T1-weighted (T1W) MRI increases adipose tissue signaling and decreases water signaling, which can show the difference between gray and white matter tissues, T2-weighted (T2W) MRI increases water signaling and Fluid Attenuated Inversion Recovery (FLAIR) images provide the clearer contours of pathological tissues, such as lesion regions. T1-weighted contrast enhance (T1CE) images is to apply contrast agent to the blood during MR. The bright area has abundant blood-brain barrier permeability supply, and the enhanced display indicates abundant blood flow. The tumor site is the site of rapid blood flow, and the T1CE images can further show the situation inside the tumor and distinguish the tumor from non-tumor lesions (that is, gangrene). Compared with other sequences (T1W, T2W, FLAIR), T1CE images can show the lesion area more clearly and directly, allowing doctors to more clearly distinguish disease between neoplastic and non-neoplastic lesions. However, the contrast agent we injected is not safe (Strijkers et al., 2007; Wahsner et al., 2018). Gadolinium-based MRI contrast agents (GBCAs) are by far the most commonly used (Hao et al., 2012). There are many adverse reactions to GBCAs, including renal and non-renal adverse reactions. Nausea, urticaria, and taste disturbance are the most common non-renal adverse events associated with GBCAs. All available GBCAs had the same incidence of these minor adverse effects (Runge, 2001). In 2008, evaluated the rate of adverse reactions to Gd-BOPTA (one of the GBCAs) in 23,553 people. This study showed that adverse reactions were similar to those of other GBCAs (Bleicher and Kanal, 2008). In Abujudeh et al. (2010) retrospectively assessed the acute adverse reaction rate of Gd-DTPA in 27,956 doses and Gd-BOPTA in 4,703 doses. The study showed that the acute adverse reaction rate of Gd-DTPA (one of the GBCAs) and Gd-BOPTA was 0.14 and 0.28% respectively (Abujudeh et al., 2010). Renal systemic fibrosis (NSF) is a serious late adverse event associated with GBCAs exposure in patients with renal insufficiency or dialysis (Thomsen, 2006). NSF is characterized by thickening, hardening and tightening of the skin with subcutaneous edema, which in severe cases leads to joint contracture and immobility. Skin changes occur primarily on the distal extremities, but may extend to the trunk. NSF may also involve the lungs, heart, liver, kidneys, skeletal muscles, diaphragm and other organs. In addition, NSF can cause death through scarring of body organs (Lin and Brown, 2007). Therefore, it is very important to find a method to generate T1CE images from other MRI sequences.

As a new field in the application of image generation technology, medical image synthesis technology aims at synthesizing target modes from one or more given modes. By applying advanced image synthesis methods, many challenging problems can be solved to a large extent. In the past few years, medical image synthesis is generally regarded as a patch-based regression task (Huynh et al., 2015; Roy et al., 2016; Zhan et al., 2022b). Recently, deep learning has shown explosive popularity in the field of medical image analysis (Tang et al., 2020, 2022; Hu L. et al., 2022; Shi et al., 2022; Sun et al., 2022; Wang et al., 2022), especially in the field of image synthesis. For example, using models of deep learning, it is possible to spontaneously achieve and complete three-dimensional shape perception (Hu et al., 2023), and to represent brain network multimodal connectivity for Alzheimer’s disease analysis using Hypergraph GANs (Pan et al., 2021). Generative artificial intelligence can be divided into four main methods [variational autoencoder (VAEs), generative adversarial network (GANs), Flow Models and Denoising Diffusion Probabilistic Models (DDPMs)] (Gong et al., 2023). In Dar et al. (2020a,b) introduced the idea of transfer learning into a cascade neural network to facilitate MRI reconstruction. Since the successful generation of adversarial networks (GAN) (Goodfellow et al., 2020), the medical image synthesis field has invested more efforts. In particular, GAN performed well in tasks including CT to MRI synthesis (Nie et al., 2017), MRI to CT synthesis (Wolterink et al., 2017, Hiasa et al., 2018), CT to PET synthesis (Bi et al., 2017), MRI to MRI synthesis (Dar et al., 2019; Yu et al., 2019; Hong et al., 2021), low dose PET to full dose PET synthesis (Xiang et al., 2017; Wang et al., 2018; Luo et al., 2022), MRI to PET synthesis (Hu S. et al., 2022) and dose estimation (Li et al., 2022; Zhan et al., 2022a) has made promising progress in the mission. Therefore, our work is also inspired by GAN’s outstanding performance. Pix2pix (Isola et al., 2017) is a GAN model, which can learn the mapping of input images to output images. Since different modalities of MR represent different clinical significance, we wondered if the effect would be better if more modalities were added to Pix2pix. Therefore, we fuse multi-dimensional modal features in the generator at different stages. For MR multimodal fusion to generate T1CE images, Chartsias et al. (2018) propose a multi-input multi-output fully convolutional neural network model for MRI synthesis, but learn one independent encoder for each input modality of our model. Olut et al. (2018) simply input pictures of two modalities as two channels and convolve them together.

Because it is difficult to generate from T1W images to T1CE images, many models are conversions between non-enhanced magnetic resonance sequences. For that, we propose a multimodal deep learning network model to generate enhanced images from non-enhanced magnetic resonance images. In our experiment, we used the BraTS2021 dataset to compare different combinations of several non-enhanced magnetic resonance sequences (T1W, T2W, flair) to generate T1CE images. After getting the trained model, we verified it with test data.

The contributions of this work are summarized as follows.

(1) We propose a generative adversarial network model framework based on pix2pix, which incorporates multi-modal information to enhance the generation of magnetic resonance images. We gradually integrate and supplement features of different modalities at the decoder level using a multi-dimensional feature fusion strategy, thus improving the performance of the model. This results in a more comprehensive feature encoding and better results compared to single-modal inputs. The effectiveness of our experiments is validated on an open dataset, outperforming the pix2pix model using single-modal inputs. Through testing, the best combination of two modal inputs T1 and T2 has been determined, with PSNR of 23.604, NMSE of 0.529, RMSE of 0.077, and SSIM of 0.859. Compared to the best model with single modal inputs (T1, T2, Flair), the evaluation indicators show better effects, i.e., lower NMSE, NRMSE and higher PSNR, SSIM.
(2) We extracted multiple sets of features from different dimensions and utilized the complementarity between high and low-dimensional features to integrate the different advantages of same-dimensional features from different modalities. The proposed Generative Adversarial Network model provides a new option and a new approach for multi-modal synthesis of enhanced magnetic resonance images. This allows for the generation of enhanced MRI images of brain gliomas after registering non-enhanced MRI images, making it possible to minimize the use of GBCA in the future and reduce its side effects, which will greatly benefit patients.

## 2 Materials and methods

In this section, we introduce in detail the multimodal generative adversarial network (multimodal GAN, MS-GAN) for MR T1CE image synthesis. Part II.-I first briefly introduces the principles of the GAN model and the task of image generation, branching image generation images. Then, sections II.-II describe the pix2pix model as a benchmark model, including the generator model structure and the discriminator network structure of pix2pix. Finally, the proposed MS-GAN generator model structure is introduced in sections II.-III, and the objective function of MA-GAN is introduced in sections II.-IV.

### 2.1 Overview

Generative adversarial networks (GANs) consist of two networks: a generator and a discriminator. The role of the generator is to produce fake images, while the discriminator’s role is to distinguish between real and fake images. Through their adversarial relationship, the discriminator continuously improves its ability to distinguish, while the generator continuously improves its ability to generate. Ultimately, when the discriminator is unable to confidently determine the authenticity of the input, we can conclude that the generator has learned the ability to produce realistic images. The discriminator takes as input real images from the dataset and images generated by the generator, and outputs a probability that the input is real data. When this input stabilizes around 0.5, we can consider the discriminator difficult to determine whether the input is true or false.

The essence of the generator is a decoder, whose input is an n-dimensional vector from the standard normal distribution, and decodes through the decoder to obtain an image with the same dimensions as the real data, that is, generated images. Once this generator is trained through adversarial means, we can freely select an n-dimensional standard normal distribution vector and decode it to obtain a new image (one that has never appeared in the dataset). The GAN model not only has a simple structure and uncomplicated principles, but also has the capability to generate images that have never appeared in the dataset. Therefore, for a long time (in fact, it can be said that until today), it has become the mainstream research/use object of generative models, and a series of variants have appeared to solve different downstream problems.

In generative tasks, there is a class of tasks called image-to-image translation. That is, the input and output are images from two different sets (designated as A and B), and we generally assume they have a corresponding relationship. For example, inputting a black and white photo (A) and outputting a colored photo (B), or inputting a contour photo (A) and outputting a color-filled photo (B), etc., the baseline model used in this article is the pix2pix model, which is designed to handle such tasks. Furthermore, the authors of pix2pix have proven the effectiveness of conditional Generative Adversarial Networks (GAN) in this type of problem through a series of experiments. In other words, pix2pix is essentially a special type of conditional GAN. From this we are inspired, whether it is possible to obtain medical images that are difficult to obtain in this way. Therefore, we conducted a series of experiments based on the pix2pix model. However, in the experimental results, the quality of the generated images was poor. We considered adding additional modalities to supplement the features encoded by the generator, and using multi-modal inputs to further generate better magnetic resonance enhancement period images.

### 2.2 Baseline

The framework of the pix2pix model consists of two main components: the generator network and the discriminator network. The generator network takes input images from the source domain and aims to generate corresponding output images in the target domain. The role of the discriminator is to judge whether the generated images are real within their corresponding receptive fields.

The generator used in the pix2pix model utilizes the classic Unet structure, but this type of model structure can only take a single image input and it is difficult to incorporate information from other modalities. In order to better judge the local parts of the image, the Pix2pix discriminator network adopts a Markov discriminator (patch GAN), which divides the image into multiple fixed-size patches and judges the truthfulness of each patch separately. The average value is then taken as the final output of the discriminator. The Markov discriminator is composed entirely of convolutional layers, and the final output is an n*n matrix, with the average value of the output matrix taken as the True/False output. Each output in the output matrix represents a receptive field in the original image, corresponding to a patch in the original image. Doing this can make the input of the discriminative network smaller, reduce the computational load, and speed up training. However, the generator part of the pix2pix model is not suitable for multi-modal input images, so we propose a generative adversarial network for multi-modal input.

**FIGURE 1:**
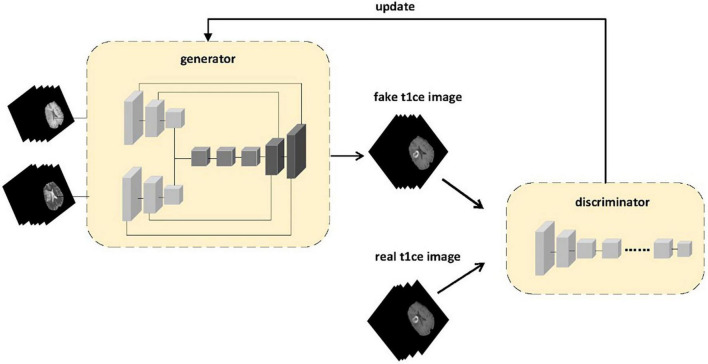
The proposed framework of multi-sequence GAN consists of a generator and a discriminator. Two different sequences of MR images are inputted into the generator. The MR images generated by the generator and the real enhanced sequence MR images are fed into the discriminator. In the discriminator model, instead of adding input contour images to the loss function as in the pix2pix model to make the generator’s predicted result match the contour of the original image, we use the generated enhanced MRI images and real enhanced MRI images as inputs to the discriminator to ensure that the generated images are more likely to resemble real enhanced MRI images.

We have improved the generator part based on the framework of pix2pix, introducing a multi-modal structure that allows the generator model to take in not only a single modality input, but also additional modalities as shown in Figure 1. For our task, we have tried different combinations of inputs, using T1, T2, and Flair sequences as inputs separately, as well as inputting combinations of T1, T2, and Flair into our generator model for comparison. We have also compared the generation effects between different imaging modalities. We will describe in detail the specific structure of the improved generator model. As for the discriminator model, we still use the Markov discriminator consistent with the pix2pix model to speed up training and obtain higher quality generated images.

### 2.3 Generator framework

**FIGURE 2:**
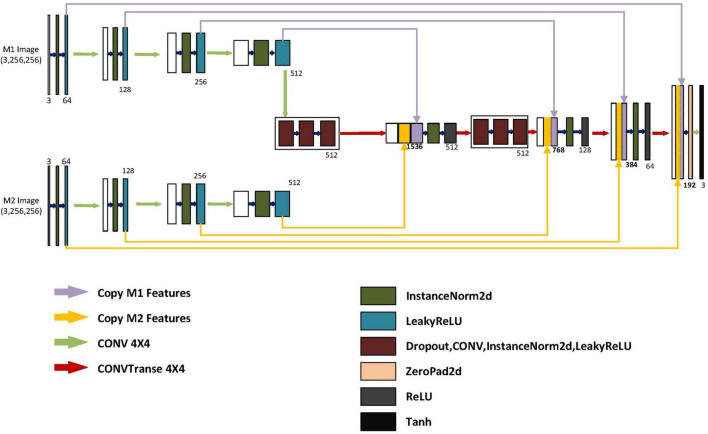
Structure of the generator. The generator includes two autoencoders and one decoder. M1 represents the input for the first class of MRI images. M2 represents the input for the second class of MRI images. The encoder for M1 and M2 is similar, with input image dimensions of 3x256x256. After the first layer of encoding, the dimensions become 64x128x128. After the second layer of encoding, the dimensions become 128x64x64. After the third layer of encoding, the dimensions become 256x32x32. After the fourth layer of encoding, the dimensions become 512x16x16. The model integrates different encoding features separately in different blocks of the decoder. After feature fusion in different layers of the decoding blocks, the dimensions are 1536X16X16, 768X32X32, and 384X64X64, respectively. The low-dimensional features of the M1 and M2 encoders and the high-dimensional features of the previous layer’s output are fused into 192X128X128 after the final fusion. Finally, after up sampling, convolutional layers, and a Tanh activation function layer, it becomes a generated image of size 3X256X256.

As shown in Figure 2, the generator model is mainly composed of three parts, namely the M1 feature extraction module, the M2 feature extraction module, and the final decoding module. The M1 and M2 feature extraction modules are the encoding parts of different modalities, each consisting of four encoding blocks, with each block containing a convolutional layer, a normalization layer, and an activation layer. Each block gradually extracts low-dimensional features to high-dimensional features from left to right and then merges these extracted four groups of features into the final part, which is the decoding module. In the decoding module, in order to integrate more detailed information from low-dimensional features and more semantic information from high-dimensional features, we merge the four different high and low-dimensional features extracted from different modalities into the four decoders of the decoding module. To prevent overfitting, we have added two sets of regularization modules, each containing dropout layers, deconvolution layers, normalization layers, and activation layers. In order to extract larger image feature information for subsequent decoding, we have used a 4x4 convolutional kernel. Because the generated results mainly depend on a specific image instance, not on processing a batch of images, batch normalization is not applicable. Instead, instance normalization normalizes within a single channel, which can accelerate model convergence and maintain independence between each image instance.

### 2.4 Objective functions

In order to consider multimodal generation, we made improvements based on pix2pix. In the discriminator model, an additional set of channels is added to input extra modes. The loss function includes the loss function of the generation model and the loss function of the discriminator model. The generator’s loss function consists of adversarial loss and pixel loss. The adversarial loss is as follows:


(1) $${ℒ}_{a\,d\,v}={𝔼}_{x\,1,y}\,\left[l\,o\,g\,D\,\left(x\,1,y\right)\right]+{𝔼}_{x\,2,y}\,\left[l\,o\,g\,D\,\left(x\,2,y\right)\right]$$

$$+{𝔼}_{x\,1,x\,2,y}\,\left[l\,o\,g\,\left(1-D\,\left(G\,\left(x\,1,x\,2\right),y\right)\right)\right]$$

The loss function for the discriminator is the adversarial loss. x1 represents the first type of magnetic resonance image input into the model, such as T1, T2, Flair; x2 represents the second type of magnetic resonance image input into the model (different from the x1 input modality); y represents the real T1CE magnetic resonance image;

As with the pix2pix model, we add the L1 loss to the generator loss. When it comes to loss selection, L1 loss is chosen instead of L2 loss to ensure less ambiguity. The pixel loss is regularized with L1 as follows:


(2) $$L_{p\,i\,x}={𝔼}_{x\,1,x\,2,y}\,\left[{||y-G\,\left(x\,1,x\,2\right)||}_{1}\right]$$

Adding the Equations 1, 2 finally gives us our final generator loss function.

Generator loss function is as follows:


(3) $$L_{G}=L_{a\,d\,v}+\lambda \,{ℒ}_{p\,i\,x}\,\left(G\right)$$

The model parameters are updated by minimizing the generated model loss function and maximizing the discriminant model loss function, where λ controls the degree of regularization.

## 3 Results

### 3.1 Datasets

The BraTS2021 (Menze et al., 2015; Bakas et al., 2017a,b; Baid et al., 2021) dataset consists of 1,254 subjects with MR images from four modalities: T1W, T2W, FLAIR, and T1CE (size: 240 × 240 × 155 voxels), along with their brain tumor segmentation labels. In this work, we performed a total of three synthesis tasks on BraTS2021:

(1) Generating T1CE from T1 and T2 images.
(2) Generating T1CE from T1 and FLAIR images.
(3) Generate T1CE from T2 and FLAIR images.
(4) Generate T1CE from T1 image.
(5) Generate T1CE from T2 image.
(6) Generate T1CE from FLAIR image.

For each synthesis task, the entire dataset is used in a five-fold cross-validation manner. That is, in each cross-validation split, four-fifths of the entire dataset make up a training set, and the remaining one-fifth make up a validation set. If most of the data is used for training, the training time is too long. When we use all subjects for training, it takes 72 h for each training session using NVIDIA A6000. We used a total of 350 subjects to train and validate our model, and 81 subjects for testing. Subsequently, lesion areas were extracted from their brain tumor segmentation labels. A total of 17,920 images were used for training, with 80% used for the training set and 20% used for the validation set to update model parameters. 5,179 images were used for the final testing of the model.

### 3.2 Experimental settings

pix2pix and our model used the same set of hyperparameters in each of their three synthetic tasks, a total of six tasks. The six tasks were trained for 120 epochs. Adam (Kingma and Ba, 2014) was used as the optimizer. To ensure the approximate shape and outline of the image, The regularization hyperparameter λ in Equation 3 was set to 100. Other hyperparameters were set to batch size 64 and input image size (256,256). Since the learning rate should decrease as we approach the global minimum of the loss value so that the model can get as close to this value as possible, we used a cosine annealing strategy to dynamically change the learning rate. In the cosine function, as × increases, the cosine value first decreases slowly, then accelerates downward, and then decreases slowly again. This downward pattern can be combined with a learning rate to produce very effective computational results.

### 3.3 Evaluation measures

PSNR (Peak Signal-to-Noise Ratio), SSIM (Structural Similarity Metric), RMSE (Root Mean Square Error), and NMSE (Normalized Mean Square Error) are commonly used to assess the quality of the resulting images. NMSE is used to assess the degree of pixel-level difference between the repaired image and the original image. The smaller the NMSE value, the more similar the images are. PSNR is an objective evaluation metric used to evaluate noise levels or image distortion, the larger the PSNR, the smaller the distortion and the better the quality of the resulting image. SSIM is used to assess the degree of similarity between two images. The value range of SSIM is 0–1, and the closer the value is to 1, the more similar the image is.

### 3.4 Results on BRATS2021

In this section, we present the experimental results of the BraTS2021 dataset. Tables 1–4, respectively report the quantitative results of the entire images for the synthesis task under different magnetic resonance modal inputs for two models, which are evaluated using NMSE, RMSE, PSNR, and SSIM. To better assess the quality of the models, we employ 5-fold cross-validation to train and test the models. Five-fold cross-validation is a commonly used machine learning model evaluation technique, typically used to estimate the performance and generalization ability of the model. The basic idea is to divide the original data set into five equally sized subsets, with four used for training the model and one used for testing the model. This process is repeated five times, each time selecting a different subset as the test set and the remaining subsets as the training set. To test the significance of baseline improvement, Dunnett test was applied to assess the statistical significance of methodological differences between subjects. Dunnett’s method is used in Analysis of Variance (ANOVA) to create confidence intervals for the difference between the mean of each factor level and the mean of the control group. The results are shown in Figure 4.

**TABLE 1:** NMSE results (our model and pix2pix) comparison.

Fold	Our model (T1 & T2)	Our model (T1 & flair)	Our model (T2 & flair)	Pix2pix (T1)	Pix2pix (T2)	Pix2pix (Flair)
1	**0.559**	0.615	0.593	0.621	0.616	0.641
2	**0.527**	0.565	**0.527**	0.562	0.551	0.557
3	**0.523**	0.569	0.533	0.570	0.544	0.570
4	**0.519**	0.562	0.541	0.608	0.542	0.559
5	**0.520**	0.560	0.538	0.568	0.551	0.585
Average	**0.529**	0.574	0.546	0.586	0.561	0.582

The bold values indicate best value in each row.

**TABLE 2:** PSNR results (our model and pix2pix) comparison.

Fold	Our model (T1 & T2)	Our model (T1 & Flair)	Our model (T2 & Flair)	Pix2pix (T1)	Pix2pix (T2)	Pix2pix (Flair)
1	**23.122**	22.424	22.695	22.085	22.173	21.817
2	**23.582**	23.105	23.561	22.867	22.962	22.885
3	**23.739**	23.047	23.475	22.823	23.169	22.739
4	**23.803**	23.141	23.441	22.270	23.119	22.891
5	**23.772**	23.180	23.420	22.828	23.011	22.603
Average	**23.604**	22.939	23.318	22.574	22.887	22.587

The bold values indicate best value in each row.

**TABLE 3:** RMSE results (our model and pix2pix) comparison.

Fold	Our model (T1 & T2)	Our model (T1 & Flair)	Our model (T2 & Flair)	Pix2pix (T1)	Pix2pix (T2)	Pix2pix (Flair)
1	**0.081**	0.089	0.085	0.089	0.088	0.091
2	**0.077**	0.082	0.077	0.082	0.081	0.081
3	**0.077**	0.083	0.078	0.083	0.080	0.083
4	**0.076**	0.081	0.079	0.088	0.079	0.082
5	**0.076**	0.082	0.079	0.083	0.081	0.085
Average	**0.077**	0.083	0.079	0.085	0.082	0.084

The bold values indicate best value in each row.

**TABLE 4:** SSIM results (our model and pix2pix) comparison.

Fold	Our model (T1 & T2)	Our model (T1 & Flair)	Our model (T2 & Flair)	Pix2pix (T1)	Pix2pix (T2)	Pix2pix (Flair)
1	0.855	**0.856**	0.854	0.843	0.847	0.838
2	0.856	**0.859**	0.860	0.847	0.850	0.849
3	**0.861**	0.859	0.860	0.853	0.855	0.848
4	**0.861**	0.860	**0.861**	0.850	0.851	0.850
5	**0.863**	0.860	0.858	0.848	0.851	0.850
Average	**0.859**	**0.859**	**0.859**	0.848	0.851	0.847

The bold values indicate best value in each row.

#### 3.4.1 Compare with baseline

In order to study the effectiveness of the proposed multi-modal fusion input, we compared our proposed multi-modal input with their corresponding baselines, i.e., the original pix2pix. Tables 1–4, respectively show the results of different evaluation metrics in the six synthesis tasks of the five-fold cross-validation. As shown in the four tables, in the comparison of the two models, the multi-modal input achieved higher PSNR and SSIM as well as lower NMSE and RMSE in the synthesis tasks than the single-modal input. The proposed method outperforms its corresponding baseline in all evaluation metrics.

As shown in Table 1, among the NMSE evaluation indicators, the input effect of T1 images is the worst in the single-modal input pix2pix model, and the input effect of T2 sequence is the best. In our proposed model, the T1 and T2 sequence inputs have the best effect, and the T1 and flair sequence inputs have the worst effect. The inputs of T1 and T2 with the best performance of the proposed model are 0.032 lower than those of the pix2pix model, and there is a significant difference between the statistical test of *p* < 0.01, as shown in Figure 4. This indicates that the stability of the image quality generated by the dual-modal input is higher than that of the single-modal input.

As shown in Table 2, in the PSNR evaluation index, the results obtained are similar to the results of the NMSE evaluation index. In the single-modal input pix2pix model, the T1 sequence input has the worst effect, and the T2 sequence input has the best effect. The inputs of T1 and T2 with the best performance of the proposed model are 0.717 higher than those of the pix2pix model, and the statistical test *p* < 0.01 has a significant difference, as shown in Figure 4. This indicates that the degree of image information loss generated by the dual-modal input is significantly lower than that of the single-modal input.

As shown in Tables 3, 4, in the RMSE evaluation index, the results obtained are similar to those of the NMSE evaluation index. In the SSIM evaluation indicators, the highest input of the T2 sequence of the pix2pix model is 0.851 in the SSIM evaluation index, but the average value of the proposed model is 0.859 after five-fold cross-validation, and the input of the three different combinations is 0.859, and the *p* < 0.01 is statistically tested, which has a significant difference. This shows that the image generated by the input of the multimodal model is closer to the image of the target T1CE sequence than that generated by the input of the single modality of the pix2pix model.

**FIGURE 3:**
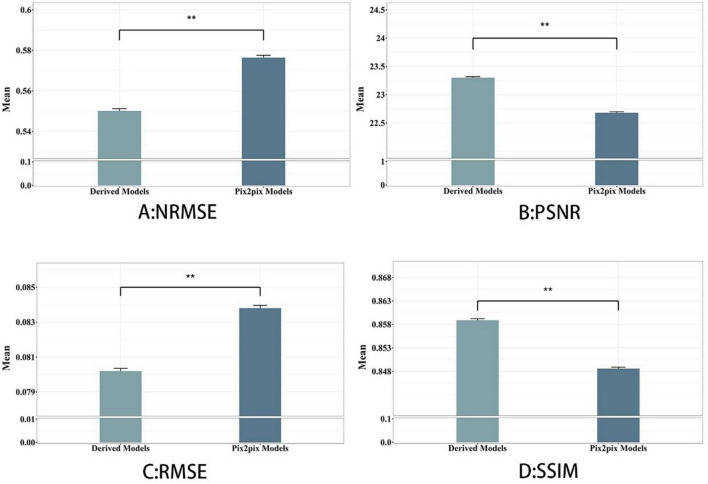
Derived model is our proposed model. We see that using hybrid input models achieves better results than single-input models. **p < 0.001, indicating strong distinctiveness.

To investigate the validity of the proposed multimodal fusion inputs, and in order to better compare with the baseline model, we compare and evaluate all the generated images obtained by a single modal input sequence (T1, T2, Flair) and all the generated images obtained by a multimodal input sequence (T1&T2, T1&Flair, T2&Flair) as a whole, as shown in Figure 3. On the Comparison of the two models, the synthesis task with multimodal input can achieve higher PSNR and SSIM and lower NMSE than the synthesis task with single modal input, and the proposed method is better than its corresponding baseline.

#### 3.4.2 Optimal magnetic resonance modal combination model

**FIGURE 4:**
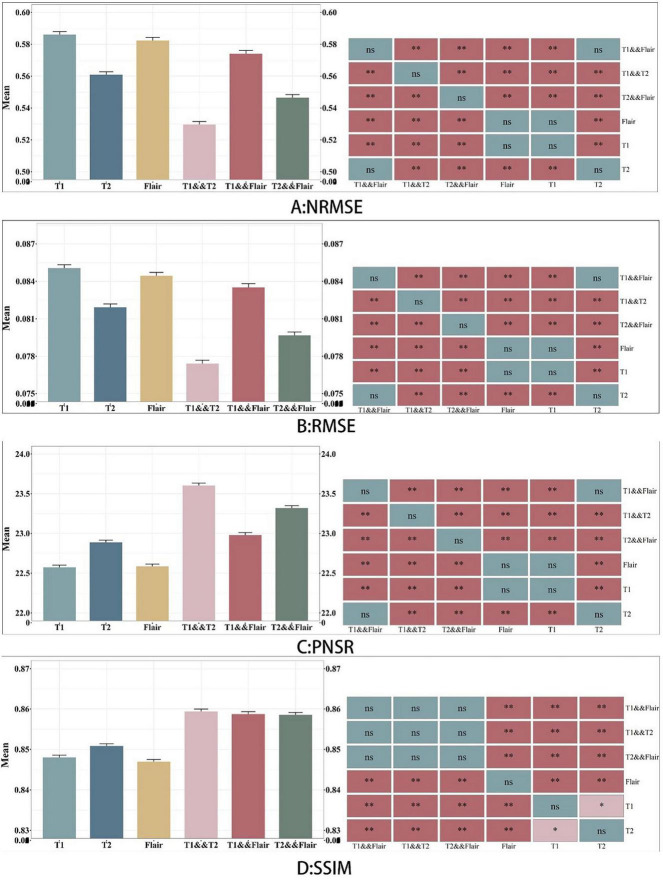
Comparison between six sets of models. A, B, C and D represents the four evaluation indicators of NMSE, RMSE, PSNR and SSIM. The figure on the left represents the average value of the test data, and the graph on the right indicates whether there is a significant difference between the models, where NS represents no significant difference.

**FIGURE 5:**
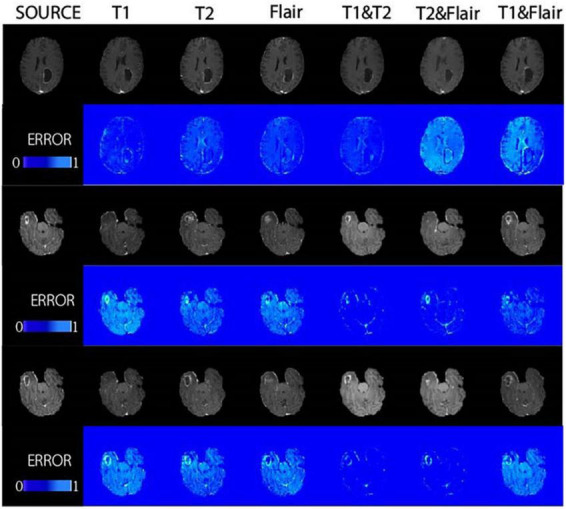
The proposed method was demonstrated in glioma patients, with images of different modalities fed for the synthesis of T1CE-weighted images in the BRATS dataset. Displays the composite results of the different inputs and their corresponding error plots, as well as the real target image (source). At the same time, the proposed method achieves reliable synthesis and significantly improves the synthesis quality of lesion areas.

In Figure 4, we found that using the combination of T1 and T2 inputs in the model achieves better results, surpassing T1&&Flair, T2&&Flair, and other single modal inputs. In the SSIM evaluation metric, the five-fold cross-validation evaluation result averages 0.859, surpassing the Pix2pix model (*p* < 0.01). In the PSNR evaluation metric, the five-fold cross-validation evaluation result averages 23.604, surpassing models with other input modalities (*p* < 0.01). In the RMSE evaluation metric, the five-fold cross-validation evaluation result averages 0.077, lower than models with other input modalities (*p* < 0.01). These results indicate that the combination of T1 and T2 inputs can achieve better image generation quality and stability. In Figure 5, we can see that the combination input of T1 and T2 results in a clearer local detail in the model’s output, and the images they generate appear to have a higher similarity to the actual situation, especially in the lesion area. Multimodal input results generally have better detail performance in the lesion area than single-modal input results.

#### 3.4.3 Clinical physician assessment for the generating test images

**FIGURE 6:**
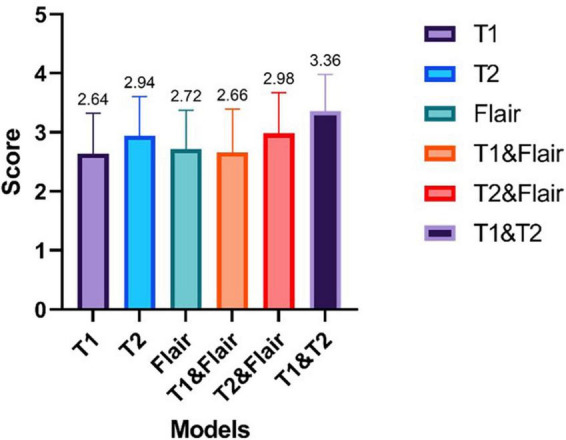
The results of enhanced magnetic resonance imaging tests generated by six different modes were evaluated by two chief radiologists with over 5 years of work experience. The scoring ranged from 1 to 4, with higher scores indicating better image quality.

We selected 53 enhanced magnetic resonance images generated in the test set for evaluation. These images were generated by six different models, including single-input sequence pix2pix models for T1, T2, and Flair, as well as our proposed models for dual-input sequences T1&&T2, T1&&Flair, and T2&&Flair. The image quality evaluation was conducted by two chief radiologists with more than 5 years of experience. The image quality was rated on a scale of 1 to 4, with higher numbers indicating better quality. The assessed images and the evaluation results for all images can be found in the Supplementary material. The evaluation results are shown in Figure 6.

From Figure 6, we can see that the average ratings of the enhanced magnetic resonance images generated from single modality input sequences T1, T2, Flair are 2.64, 2.94, and 2.72, respectively. The pix2pix model’s average rating for the single input model is 2.73. The average ratings of the enhanced magnetic resonance images generated from the dual modality input sequences T1&Flair, T2&Flair, T1&T2 are 2.66, 2.98, and 3.36, respectively. The average rating of our proposed dual modality input model is 3, which is 0.27 higher than the single input model. In the T1&T2 bimodal input model, the average score is higher than 3, at 3.36. This is 0.42 higher than the highest model in the single sequence input model (T2).

## 4 Discussion

In the BraTS2021 dataset, we proposed a method for synthesizing enhanced MRI images based on non-enhanced MRI images. Compared with the classical architecture pix2pix model, our method demonstrated that by inputting MRI images of various different modalities, richer image features can be extracted to generate images that are closer to the target enhanced images, with higher image quality and generation stability. Additionally, better results were achieved in the generation of lesions in the specific region. Furthermore, we explored the effects of generating MRI images of various modalities based on this method.

This model makes it possible to obtain MRI enhanced images in the future without the use of contrast agents, thus avoiding some of the adverse reactions caused by contrast agents. In this work, we propose a multi-modal GAN-based model aimed at synthesizing multi-modal input MR images. In addition to the existing single-modal pix2pix model, our proposed model also has an additional modal input to learn multi-modal image feature mappings. This enables our model to flexibly and comprehensively handle anatomical structures and lesion areas for better synthesis. The effectiveness of the proposed strategy can be demonstrated by the advantage of the model over its respective baseline (pix2pix). Through our performed experiments comparing single-modal input and multi-modal input, we have learned the importance of multi-modal input for the results. This is because, in the generator, we are given different modal MRI image inputs, and use the encoder to extract low-dimensional features to high-dimensional features in four different sets of dimensions. The low-dimensional features have higher resolution, contain more positional and detailed information, but due to fewer convolutions, their semantic content is lower and they have more noise. High-dimensional features contain stronger semantic information, but have low resolution and poor perception of details, so we extracted multiple sets of features of different dimensions. By utilizing the complementarity between high and low-dimensional features, and integrating the advantages of different modalities under the same dimension, we adopted a multi-dimensional feature fusion strategy. In the decoder, different modalities’ features are gradually and hierarchically fused to enhance the performance of the model, making the encoded features more comprehensive, and thus yielding better results compared to single-modality inputs. At the same time, according to the test experiments including the calculation of image quality evaluation indicators such as PSNR and SSIM, as well as the evaluation by actual radiologists, we found that for the calculated image quality evaluation indicators such as SSIM and PSNR, the effect of the bimodal model is better than the unimodal model. For the evaluation by actual radiologists, the bimodal models T1&Flair and T2&Flair did not have better effects in actual clinical applications, but for the T1&T2 bimodal model, its effect is better than other sequence input models. Through the above experiments, we have determined the optimal combination of two modal inputs, T1 and T2, providing new choices and new ideas for cross-modal synthesis of MR images. Through the above experiments, we have identified the best combination of two modal inputs T1 and T2, providing new options and new ideas for cross-modal synthesis of MR images.

Our work has some limitations. First, due to the performance issues of GPU, it is not feasible to include all the data from the BraTS2021 dataset to make the results more reliable. Additionally, the use of 2D networks ignores some spatial features of the 3D images. Second, while the use of multimodal inputs does improve the generation of enhanced magnetic resonance images compared to single modal inputs, the improvement is small. Furthermore, due to limitations of the generative adversarial network itself, the quality of the generated images is uneven and does not reliably generate enhanced phase images. Finally, due to the pre-processing of the BraTS2021 dataset we used, our method may be limited in actual clinical applications. Therefore, in the future, our work can be further improved in the following aspects. First, due to the limited memory size of a single GPU card, the use of input with two-dimensional medical images ignores some spatial features of the three-dimensional space. In our future work, we will explore the use of more GPU cards to achieve better generation results for three-dimensional images. Secondly, the generative adversarial networks currently used in our work are not suitable for widespread application in stable generation-enhanced MRI images due to some inherent characteristics. And the assessment method has certain problems. Although we scored very high in overall measurement, we may not have done well in enhancing contrast estimation methods. However, we selected 50 images for actual clinical assessment by doctors to make up for this deficiency. In our future work, we will explore the field of diffusion models (such as applying multimodal generation to stable diffusion models) to bridge the domain gap, thereby training models to achieve better results. Finally, in order to better serve actual clinical work, We will establish our own clinical dataset to build better models, so that the model can be more widely applied to various tumors, rather than just gliomas.

In conclusion, this article proposes a new model based on pix2pix for generating multi-modal input for T1CE MR image synthesis. The experimental results demonstrate that our sample multi-modal input significantly improves the performance of the pix2pix model and outperforms the pix2pix method in multiple MR image synthesis tasks. Furthermore, the optimal combination of modalities T1 and T2 is obtained for generating T1CE images with multi-modal input. Although this model suffers from issues related to stability, this allows for the generation of enhanced MRI images of brain gliomas after registering non-enhanced MRI images, making it possible to minimize the use of GBCA in the future and reduce its side effects, which will greatly benefit patients.

## Data availability statement

The public dataset (BraTs2021) that support the findings of this study are available at https://ui.adsabs.harvard.edu/abs/2021arXiv210702314B.

## Ethics statement

Ethical review and approval was not required for the studies involving humans because the dataset used is a public dataset (BraTS2021), and references to the dataset have been added (Menze et al., 2015; Bakas et al., 2017a,b; Baid et al., 2021). The studies were conducted in accordance with the local legislation and institutional requirements. Written informed consent for participation was not required from the participants or the participants’ legal guardians/next of kin in accordance with the national legislation and institutional requirements.

## Author contributions

LL: Conceptualization, Methodology, Software, Writing – original draft, Writing – review and editing. JY: Methodology, Writing – original draft, Writing – review and editing. YL: Formal analysis, Validation, Writing – review and editing. JW: Data curation, Validation, Writing – original draft. RF: Funding acquisition, Writing – review and editing. DW: Supervision, Writing – review and editing. YY: Funding acquisition, Writing – review and editing.
