# Supplementary figures and images for: Multi-sequence generative adversarial network: better generation for enhanced magnetic resonance imaging images (part 2 of 2)
Source: Front Comput Neurosci. 2024 May 22;18:1365238. doi: 10.3389/fncom.2024.1365238 (PMC11151883; doi:10.3389/fncom.2024.1365238)

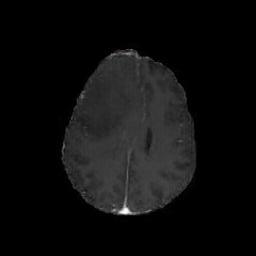

Supplement: Supplementary file 1 [file Data_Sheet_1.ZIP › Supplementary materials/evaluate/BraTS2021_00507_91_t1ce/BraTS2021_00507_91_t1ce_t1.jpg]

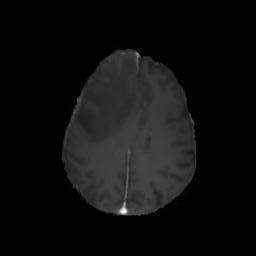

Supplement: Supplementary file 1 [file Data_Sheet_1.ZIP › Supplementary materials/evaluate/BraTS2021_00507_91_t1ce/BraTS2021_00507_91_t1ce_t1_flair.jpg]

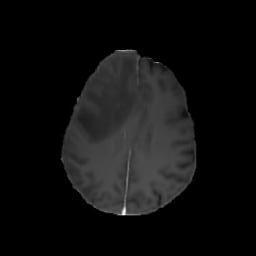

Supplement: Supplementary file 1 [file Data_Sheet_1.ZIP › Supplementary materials/evaluate/BraTS2021_00507_91_t1ce/BraTS2021_00507_91_t1ce_t1_t2.jpg]

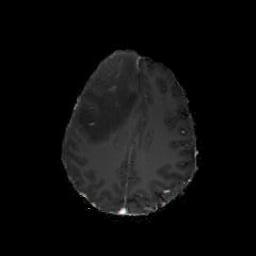

Supplement: Supplementary file 1 [file Data_Sheet_1.ZIP › Supplementary materials/evaluate/BraTS2021_00507_91_t1ce/BraTS2021_00507_91_t1ce_t2.jpg]

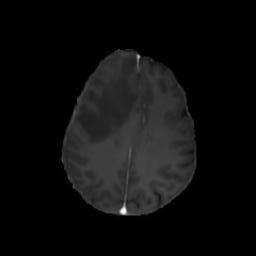

Supplement: Supplementary file 1 [file Data_Sheet_1.ZIP › Supplementary materials/evaluate/BraTS2021_00507_91_t1ce/BraTS2021_00507_91_t1ce_t2_flair.jpg]

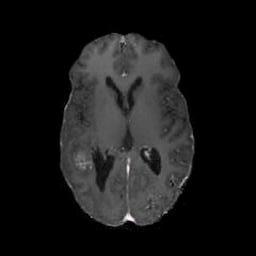

Supplement: Supplementary file 1 [file Data_Sheet_1.ZIP › Supplementary materials/evaluate/BraTS2021_00510_70_t1ce/BraTS2021_00510_70_t1ce_flair.jpg]

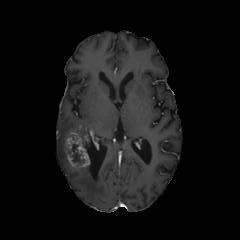

Supplement: Supplementary file 1 [file Data_Sheet_1.ZIP › Supplementary materials/evaluate/BraTS2021_00510_70_t1ce/BraTS2021_00510_70_t1ce_real.jpg]

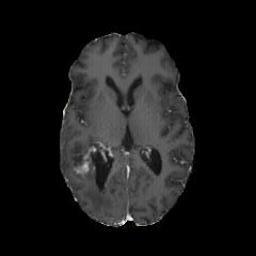

Supplement: Supplementary file 1 [file Data_Sheet_1.ZIP › Supplementary materials/evaluate/BraTS2021_00510_70_t1ce/BraTS2021_00510_70_t1ce_t1.jpg]

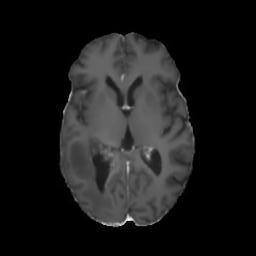

Supplement: Supplementary file 1 [file Data_Sheet_1.ZIP › Supplementary materials/evaluate/BraTS2021_00510_70_t1ce/BraTS2021_00510_70_t1ce_t1_flair.jpg]

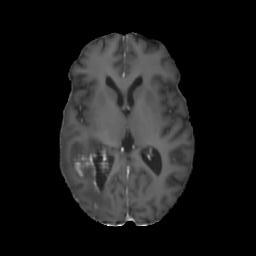

Supplement: Supplementary file 1 [file Data_Sheet_1.ZIP › Supplementary materials/evaluate/BraTS2021_00510_70_t1ce/BraTS2021_00510_70_t1ce_t1_t2.jpg]

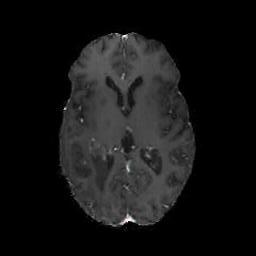

Supplement: Supplementary file 1 [file Data_Sheet_1.ZIP › Supplementary materials/evaluate/BraTS2021_00510_70_t1ce/BraTS2021_00510_70_t1ce_t2.jpg]

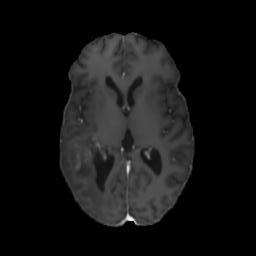

Supplement: Supplementary file 1 [file Data_Sheet_1.ZIP › Supplementary materials/evaluate/BraTS2021_00510_70_t1ce/BraTS2021_00510_70_t1ce_t2_flair.jpg]

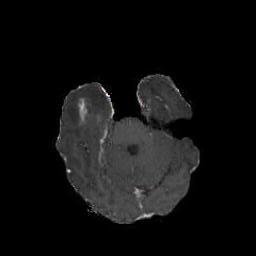

Supplement: Supplementary file 1 [file Data_Sheet_1.ZIP › Supplementary materials/evaluate/BraTS2021_00511_36_t1ce/BraTS2021_00511_36_t1ce_flair.jpg]

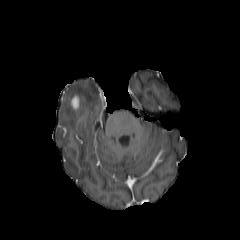

Supplement: Supplementary file 1 [file Data_Sheet_1.ZIP › Supplementary materials/evaluate/BraTS2021_00511_36_t1ce/BraTS2021_00511_36_t1ce_real.jpg]

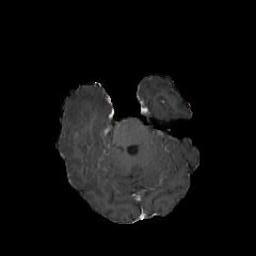

Supplement: Supplementary file 1 [file Data_Sheet_1.ZIP › Supplementary materials/evaluate/BraTS2021_00511_36_t1ce/BraTS2021_00511_36_t1ce_t1.jpg]

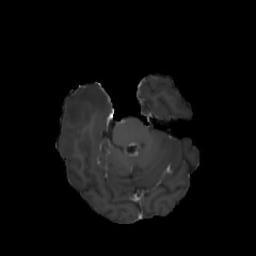

Supplement: Supplementary file 1 [file Data_Sheet_1.ZIP › Supplementary materials/evaluate/BraTS2021_00511_36_t1ce/BraTS2021_00511_36_t1ce_t1_flair.jpg]

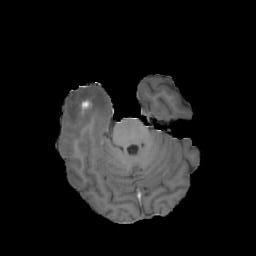

Supplement: Supplementary file 1 [file Data_Sheet_1.ZIP › Supplementary materials/evaluate/BraTS2021_00511_36_t1ce/BraTS2021_00511_36_t1ce_t1_t2.jpg]

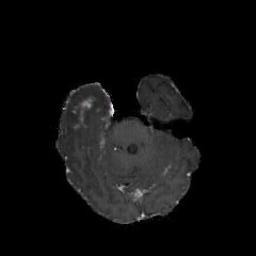

Supplement: Supplementary file 1 [file Data_Sheet_1.ZIP › Supplementary materials/evaluate/BraTS2021_00511_36_t1ce/BraTS2021_00511_36_t1ce_t2.jpg]

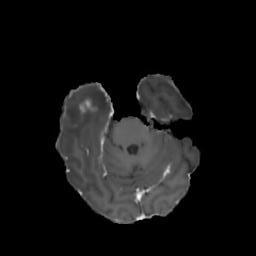

Supplement: Supplementary file 1 [file Data_Sheet_1.ZIP › Supplementary materials/evaluate/BraTS2021_00511_36_t1ce/BraTS2021_00511_36_t1ce_t2_flair.jpg]

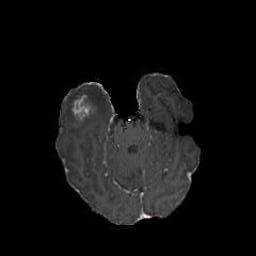

Supplement: Supplementary file 1 [file Data_Sheet_1.ZIP › Supplementary materials/evaluate/BraTS2021_00511_38_t1ce/BraTS2021_00511_38_t1ce_flair.jpg]

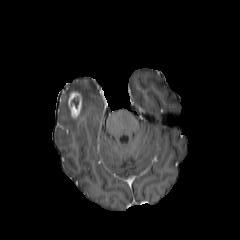

Supplement: Supplementary file 1 [file Data_Sheet_1.ZIP › Supplementary materials/evaluate/BraTS2021_00511_38_t1ce/BraTS2021_00511_38_t1ce_real.jpg]

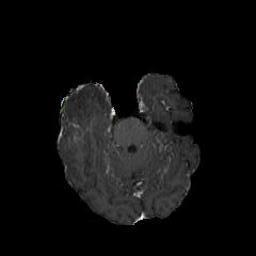

Supplement: Supplementary file 1 [file Data_Sheet_1.ZIP › Supplementary materials/evaluate/BraTS2021_00511_38_t1ce/BraTS2021_00511_38_t1ce_t1.jpg]

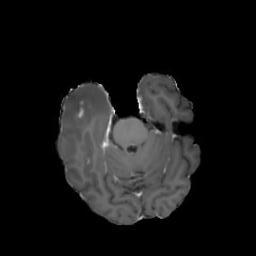

Supplement: Supplementary file 1 [file Data_Sheet_1.ZIP › Supplementary materials/evaluate/BraTS2021_00511_38_t1ce/BraTS2021_00511_38_t1ce_t1_flair.jpg]

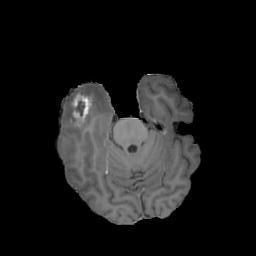

Supplement: Supplementary file 1 [file Data_Sheet_1.ZIP › Supplementary materials/evaluate/BraTS2021_00511_38_t1ce/BraTS2021_00511_38_t1ce_t1_t2.jpg]

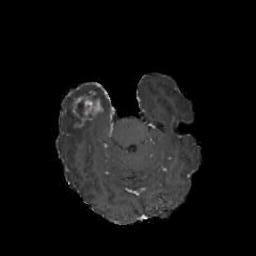

Supplement: Supplementary file 1 [file Data_Sheet_1.ZIP › Supplementary materials/evaluate/BraTS2021_00511_38_t1ce/BraTS2021_00511_38_t1ce_t2.jpg]

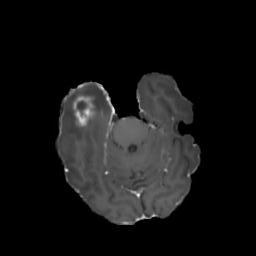

Supplement: Supplementary file 1 [file Data_Sheet_1.ZIP › Supplementary materials/evaluate/BraTS2021_00511_38_t1ce/BraTS2021_00511_38_t1ce_t2_flair.jpg]

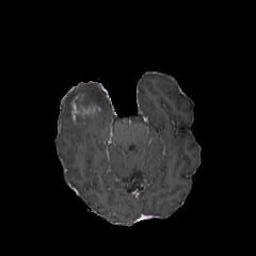

Supplement: Supplementary file 1 [file Data_Sheet_1.ZIP › Supplementary materials/evaluate/BraTS2021_00511_40_t1ce/BraTS2021_00511_40_t1ce_flair.jpg]

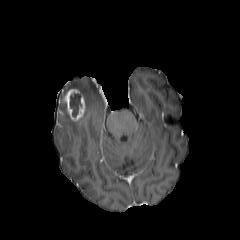

Supplement: Supplementary file 1 [file Data_Sheet_1.ZIP › Supplementary materials/evaluate/BraTS2021_00511_40_t1ce/BraTS2021_00511_40_t1ce_real.jpg]

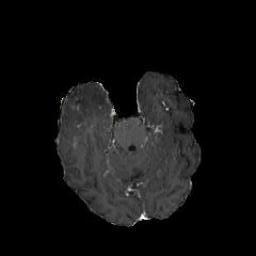

Supplement: Supplementary file 1 [file Data_Sheet_1.ZIP › Supplementary materials/evaluate/BraTS2021_00511_40_t1ce/BraTS2021_00511_40_t1ce_t1.jpg]

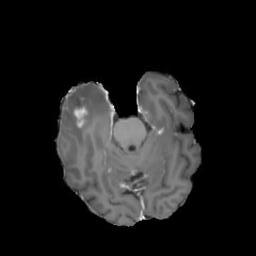

Supplement: Supplementary file 1 [file Data_Sheet_1.ZIP › Supplementary materials/evaluate/BraTS2021_00511_40_t1ce/BraTS2021_00511_40_t1ce_t1_flair.jpg]

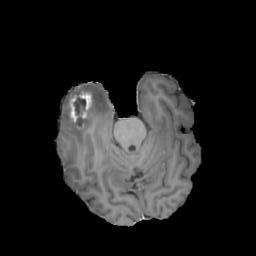

Supplement: Supplementary file 1 [file Data_Sheet_1.ZIP › Supplementary materials/evaluate/BraTS2021_00511_40_t1ce/BraTS2021_00511_40_t1ce_t1_t2.jpg]

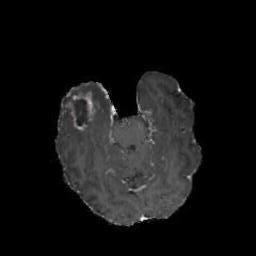

Supplement: Supplementary file 1 [file Data_Sheet_1.ZIP › Supplementary materials/evaluate/BraTS2021_00511_40_t1ce/BraTS2021_00511_40_t1ce_t2.jpg]

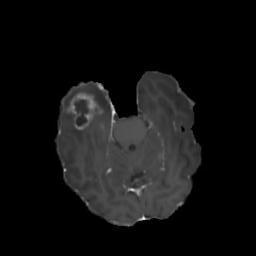

Supplement: Supplementary file 1 [file Data_Sheet_1.ZIP › Supplementary materials/evaluate/BraTS2021_00511_40_t1ce/BraTS2021_00511_40_t1ce_t2_flair.jpg]

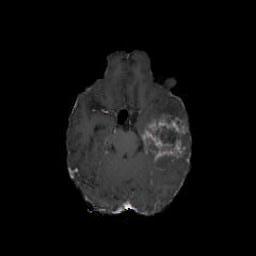

Supplement: Supplementary file 1 [file Data_Sheet_1.ZIP › Supplementary materials/evaluate/BraTS2021_00512_51_t1ce/BraTS2021_00512_51_t1ce_flair.jpg]

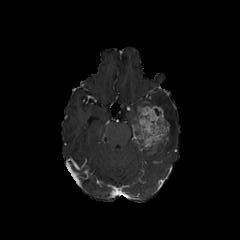

Supplement: Supplementary file 1 [file Data_Sheet_1.ZIP › Supplementary materials/evaluate/BraTS2021_00512_51_t1ce/BraTS2021_00512_51_t1ce_real.jpg]

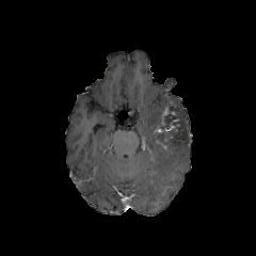

Supplement: Supplementary file 1 [file Data_Sheet_1.ZIP › Supplementary materials/evaluate/BraTS2021_00512_51_t1ce/BraTS2021_00512_51_t1ce_t1.jpg]

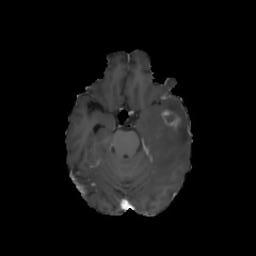

Supplement: Supplementary file 1 [file Data_Sheet_1.ZIP › Supplementary materials/evaluate/BraTS2021_00512_51_t1ce/BraTS2021_00512_51_t1ce_t1_flair.jpg]

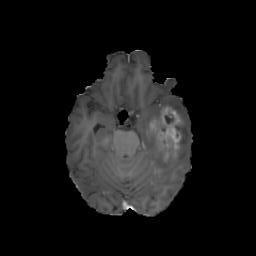

Supplement: Supplementary file 1 [file Data_Sheet_1.ZIP › Supplementary materials/evaluate/BraTS2021_00512_51_t1ce/BraTS2021_00512_51_t1ce_t1_t2.jpg]

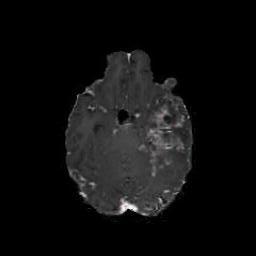

Supplement: Supplementary file 1 [file Data_Sheet_1.ZIP › Supplementary materials/evaluate/BraTS2021_00512_51_t1ce/BraTS2021_00512_51_t1ce_t2.jpg]

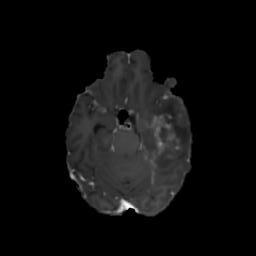

Supplement: Supplementary file 1 [file Data_Sheet_1.ZIP › Supplementary materials/evaluate/BraTS2021_00512_51_t1ce/BraTS2021_00512_51_t1ce_t2_flair.jpg]

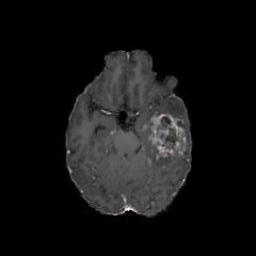

Supplement: Supplementary file 1 [file Data_Sheet_1.ZIP › Supplementary materials/evaluate/BraTS2021_00512_52_t1ce/BraTS2021_00512_52_t1ce_flair.jpg]

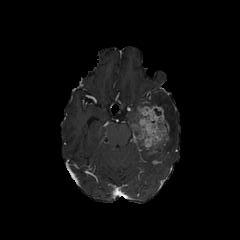

Supplement: Supplementary file 1 [file Data_Sheet_1.ZIP › Supplementary materials/evaluate/BraTS2021_00512_52_t1ce/BraTS2021_00512_52_t1ce_real.jpg]

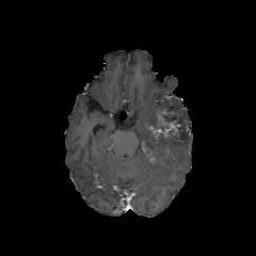

Supplement: Supplementary file 1 [file Data_Sheet_1.ZIP › Supplementary materials/evaluate/BraTS2021_00512_52_t1ce/BraTS2021_00512_52_t1ce_t1.jpg]

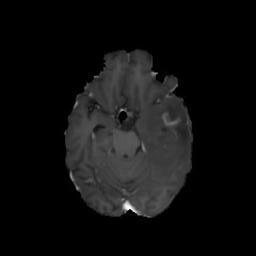

Supplement: Supplementary file 1 [file Data_Sheet_1.ZIP › Supplementary materials/evaluate/BraTS2021_00512_52_t1ce/BraTS2021_00512_52_t1ce_t1_flair.jpg]

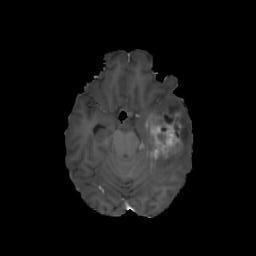

Supplement: Supplementary file 1 [file Data_Sheet_1.ZIP › Supplementary materials/evaluate/BraTS2021_00512_52_t1ce/BraTS2021_00512_52_t1ce_t1_t2.jpg]

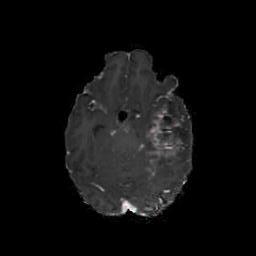

Supplement: Supplementary file 1 [file Data_Sheet_1.ZIP › Supplementary materials/evaluate/BraTS2021_00512_52_t1ce/BraTS2021_00512_52_t1ce_t2.jpg]

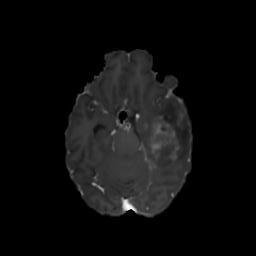

Supplement: Supplementary file 1 [file Data_Sheet_1.ZIP › Supplementary materials/evaluate/BraTS2021_00512_52_t1ce/BraTS2021_00512_52_t1ce_t2_flair.jpg]

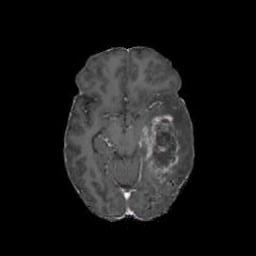

Supplement: Supplementary file 1 [file Data_Sheet_1.ZIP › Supplementary materials/evaluate/BraTS2021_00512_58_t1ce/BraTS2021_00512_58_t1ce_flair.jpg]

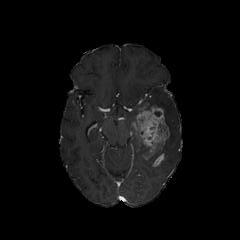

Supplement: Supplementary file 1 [file Data_Sheet_1.ZIP › Supplementary materials/evaluate/BraTS2021_00512_58_t1ce/BraTS2021_00512_58_t1ce_real.jpg]

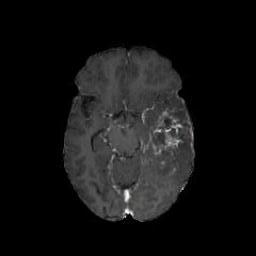

Supplement: Supplementary file 1 [file Data_Sheet_1.ZIP › Supplementary materials/evaluate/BraTS2021_00512_58_t1ce/BraTS2021_00512_58_t1ce_t1.jpg]

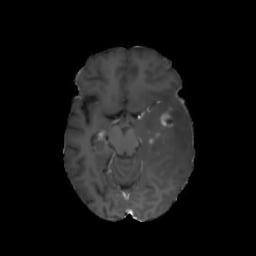

Supplement: Supplementary file 1 [file Data_Sheet_1.ZIP › Supplementary materials/evaluate/BraTS2021_00512_58_t1ce/BraTS2021_00512_58_t1ce_t1_flair.jpg]

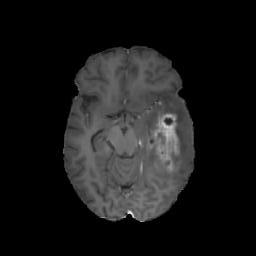

Supplement: Supplementary file 1 [file Data_Sheet_1.ZIP › Supplementary materials/evaluate/BraTS2021_00512_58_t1ce/BraTS2021_00512_58_t1ce_t1_t2.jpg]

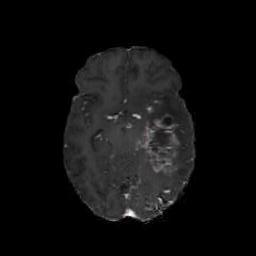

Supplement: Supplementary file 1 [file Data_Sheet_1.ZIP › Supplementary materials/evaluate/BraTS2021_00512_58_t1ce/BraTS2021_00512_58_t1ce_t2.jpg]

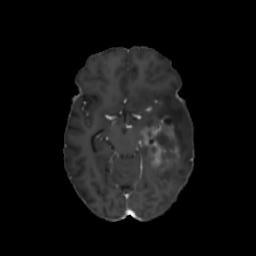

Supplement: Supplementary file 1 [file Data_Sheet_1.ZIP › Supplementary materials/evaluate/BraTS2021_00512_58_t1ce/BraTS2021_00512_58_t1ce_t2_flair.jpg]

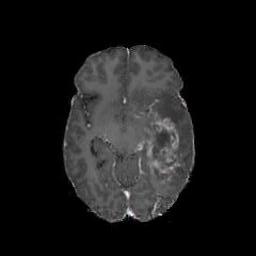

Supplement: Supplementary file 1 [file Data_Sheet_1.ZIP › Supplementary materials/evaluate/BraTS2021_00512_60_t1ce/BraTS2021_00512_60_t1ce_flair.jpg]

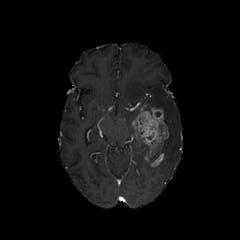

Supplement: Supplementary file 1 [file Data_Sheet_1.ZIP › Supplementary materials/evaluate/BraTS2021_00512_60_t1ce/BraTS2021_00512_60_t1ce_real.jpg]

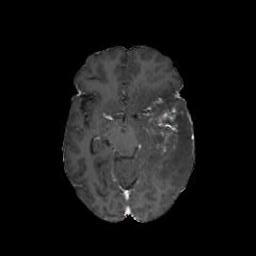

Supplement: Supplementary file 1 [file Data_Sheet_1.ZIP › Supplementary materials/evaluate/BraTS2021_00512_60_t1ce/BraTS2021_00512_60_t1ce_t1.jpg]

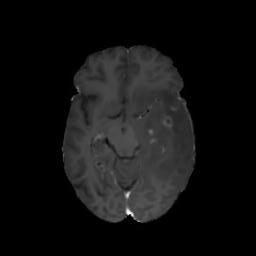

Supplement: Supplementary file 1 [file Data_Sheet_1.ZIP › Supplementary materials/evaluate/BraTS2021_00512_60_t1ce/BraTS2021_00512_60_t1ce_t1_flair.jpg]

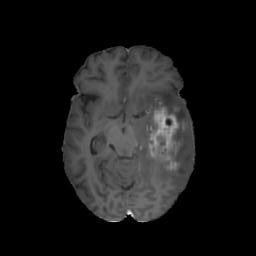

Supplement: Supplementary file 1 [file Data_Sheet_1.ZIP › Supplementary materials/evaluate/BraTS2021_00512_60_t1ce/BraTS2021_00512_60_t1ce_t1_t2.jpg]

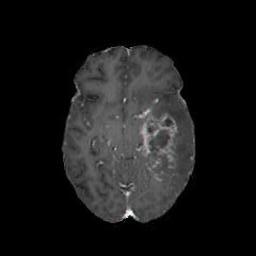

Supplement: Supplementary file 1 [file Data_Sheet_1.ZIP › Supplementary materials/evaluate/BraTS2021_00512_60_t1ce/BraTS2021_00512_60_t1ce_t2.jpg]

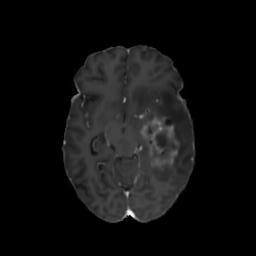

Supplement: Supplementary file 1 [file Data_Sheet_1.ZIP › Supplementary materials/evaluate/BraTS2021_00512_60_t1ce/BraTS2021_00512_60_t1ce_t2_flair.jpg]

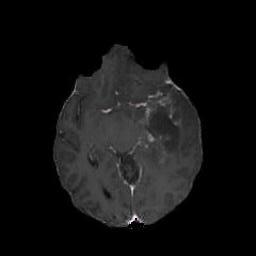

Supplement: Supplementary file 1 [file Data_Sheet_1.ZIP › Supplementary materials/evaluate/BraTS2021_00513_58_t1ce/BraTS2021_00513_58_t1ce_flair.jpg]

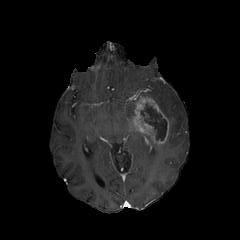

Supplement: Supplementary file 1 [file Data_Sheet_1.ZIP › Supplementary materials/evaluate/BraTS2021_00513_58_t1ce/BraTS2021_00513_58_t1ce_real.jpg]

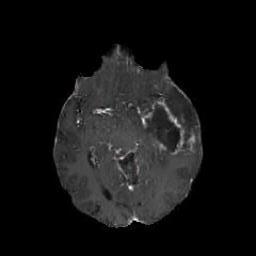

Supplement: Supplementary file 1 [file Data_Sheet_1.ZIP › Supplementary materials/evaluate/BraTS2021_00513_58_t1ce/BraTS2021_00513_58_t1ce_t1.jpg]

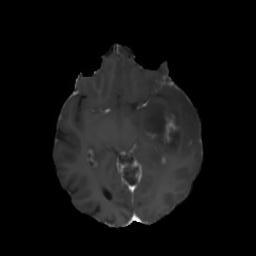

Supplement: Supplementary file 1 [file Data_Sheet_1.ZIP › Supplementary materials/evaluate/BraTS2021_00513_58_t1ce/BraTS2021_00513_58_t1ce_t1_flair.jpg]

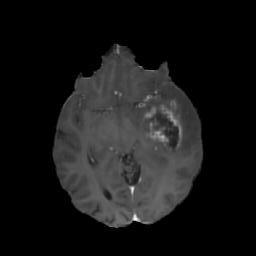

Supplement: Supplementary file 1 [file Data_Sheet_1.ZIP › Supplementary materials/evaluate/BraTS2021_00513_58_t1ce/BraTS2021_00513_58_t1ce_t1_t2.jpg]

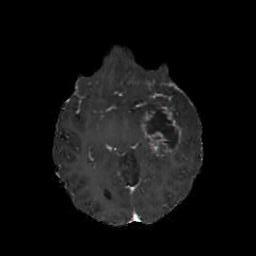

Supplement: Supplementary file 1 [file Data_Sheet_1.ZIP › Supplementary materials/evaluate/BraTS2021_00513_58_t1ce/BraTS2021_00513_58_t1ce_t2.jpg]

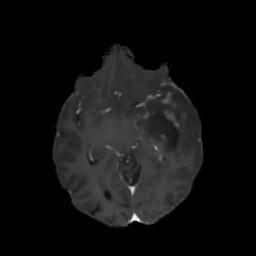

Supplement: Supplementary file 1 [file Data_Sheet_1.ZIP › Supplementary materials/evaluate/BraTS2021_00513_58_t1ce/BraTS2021_00513_58_t1ce_t2_flair.jpg]

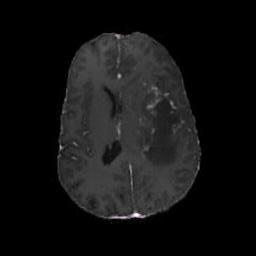

Supplement: Supplementary file 1 [file Data_Sheet_1.ZIP › Supplementary materials/evaluate/BraTS2021_00513_82_t1ce/BraTS2021_00513_82_t1ce_flair.jpg]

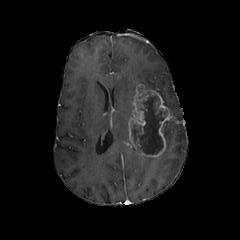

Supplement: Supplementary file 1 [file Data_Sheet_1.ZIP › Supplementary materials/evaluate/BraTS2021_00513_82_t1ce/BraTS2021_00513_82_t1ce_real.jpg]

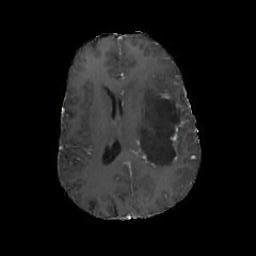

Supplement: Supplementary file 1 [file Data_Sheet_1.ZIP › Supplementary materials/evaluate/BraTS2021_00513_82_t1ce/BraTS2021_00513_82_t1ce_t1.jpg]

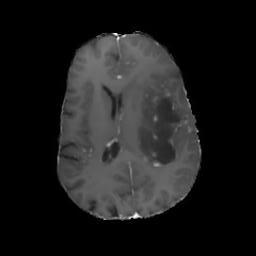

Supplement: Supplementary file 1 [file Data_Sheet_1.ZIP › Supplementary materials/evaluate/BraTS2021_00513_82_t1ce/BraTS2021_00513_82_t1ce_t1_flair.jpg]

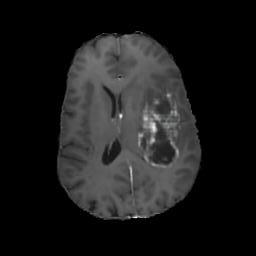

Supplement: Supplementary file 1 [file Data_Sheet_1.ZIP › Supplementary materials/evaluate/BraTS2021_00513_82_t1ce/BraTS2021_00513_82_t1ce_t1_t2.jpg]

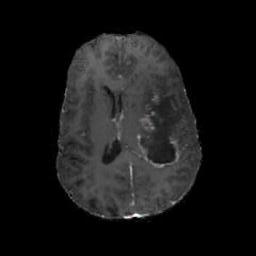

Supplement: Supplementary file 1 [file Data_Sheet_1.ZIP › Supplementary materials/evaluate/BraTS2021_00513_82_t1ce/BraTS2021_00513_82_t1ce_t2.jpg]

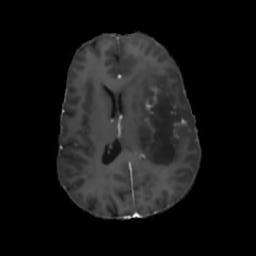

Supplement: Supplementary file 1 [file Data_Sheet_1.ZIP › Supplementary materials/evaluate/BraTS2021_00513_82_t1ce/BraTS2021_00513_82_t1ce_t2_flair.jpg]

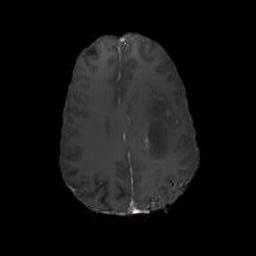

Supplement: Supplementary file 1 [file Data_Sheet_1.ZIP › Supplementary materials/evaluate/BraTS2021_00513_94_t1ce/BraTS2021_00513_94_t1ce_flair.jpg]

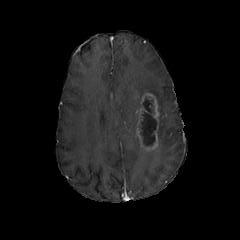

Supplement: Supplementary file 1 [file Data_Sheet_1.ZIP › Supplementary materials/evaluate/BraTS2021_00513_94_t1ce/BraTS2021_00513_94_t1ce_real.jpg]

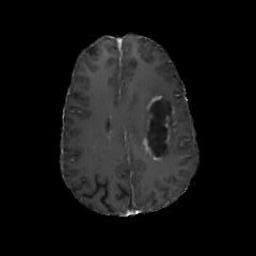

Supplement: Supplementary file 1 [file Data_Sheet_1.ZIP › Supplementary materials/evaluate/BraTS2021_00513_94_t1ce/BraTS2021_00513_94_t1ce_t1.jpg]

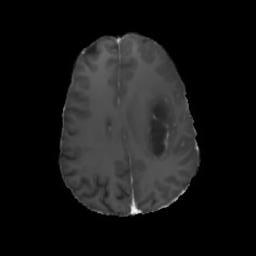

Supplement: Supplementary file 1 [file Data_Sheet_1.ZIP › Supplementary materials/evaluate/BraTS2021_00513_94_t1ce/BraTS2021_00513_94_t1ce_t1_flair.jpg]

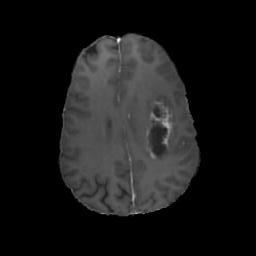

Supplement: Supplementary file 1 [file Data_Sheet_1.ZIP › Supplementary materials/evaluate/BraTS2021_00513_94_t1ce/BraTS2021_00513_94_t1ce_t1_t2.jpg]

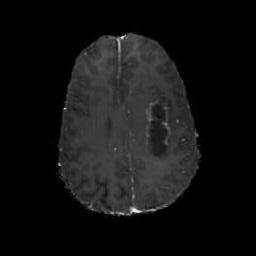

Supplement: Supplementary file 1 [file Data_Sheet_1.ZIP › Supplementary materials/evaluate/BraTS2021_00513_94_t1ce/BraTS2021_00513_94_t1ce_t2.jpg]

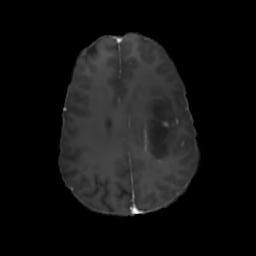

Supplement: Supplementary file 1 [file Data_Sheet_1.ZIP › Supplementary materials/evaluate/BraTS2021_00513_94_t1ce/BraTS2021_00513_94_t1ce_t2_flair.jpg]

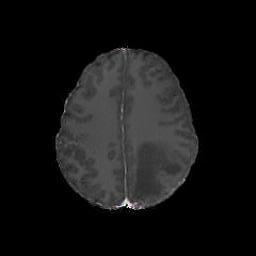

Supplement: Supplementary file 1 [file Data_Sheet_1.ZIP › Supplementary materials/evaluate/BraTS2021_00517_106_t1ce/BraTS2021_00517_106_t1ce_flair.jpg]

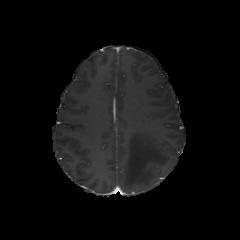

Supplement: Supplementary file 1 [file Data_Sheet_1.ZIP › Supplementary materials/evaluate/BraTS2021_00517_106_t1ce/BraTS2021_00517_106_t1ce_real.jpg]

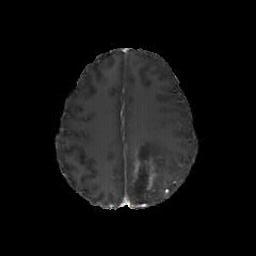

Supplement: Supplementary file 1 [file Data_Sheet_1.ZIP › Supplementary materials/evaluate/BraTS2021_00517_106_t1ce/BraTS2021_00517_106_t1ce_t1.jpg]

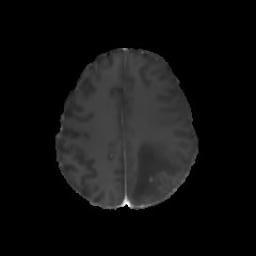

Supplement: Supplementary file 1 [file Data_Sheet_1.ZIP › Supplementary materials/evaluate/BraTS2021_00517_106_t1ce/BraTS2021_00517_106_t1ce_t1_flair.jpg]

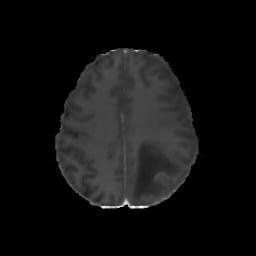

Supplement: Supplementary file 1 [file Data_Sheet_1.ZIP › Supplementary materials/evaluate/BraTS2021_00517_106_t1ce/BraTS2021_00517_106_t1ce_t1_t2.jpg]

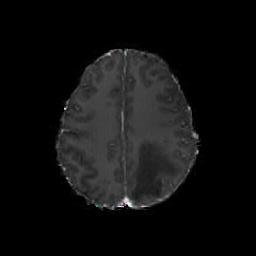

Supplement: Supplementary file 1 [file Data_Sheet_1.ZIP › Supplementary materials/evaluate/BraTS2021_00517_106_t1ce/BraTS2021_00517_106_t1ce_t2.jpg]

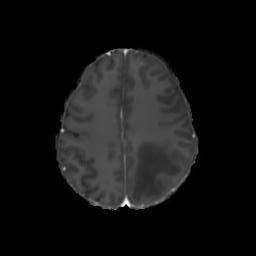

Supplement: Supplementary file 1 [file Data_Sheet_1.ZIP › Supplementary materials/evaluate/BraTS2021_00517_106_t1ce/BraTS2021_00517_106_t1ce_t2_flair.jpg]

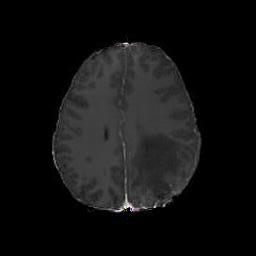

Supplement: Supplementary file 1 [file Data_Sheet_1.ZIP › Supplementary materials/evaluate/BraTS2021_00517_99_t1ce/BraTS2021_00517_99_t1ce_flair.jpg]

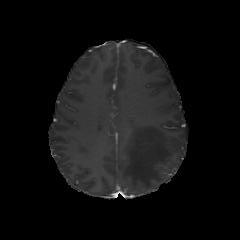

Supplement: Supplementary file 1 [file Data_Sheet_1.ZIP › Supplementary materials/evaluate/BraTS2021_00517_99_t1ce/BraTS2021_00517_99_t1ce_real.jpg]

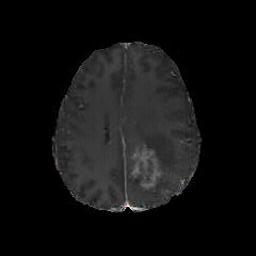

Supplement: Supplementary file 1 [file Data_Sheet_1.ZIP › Supplementary materials/evaluate/BraTS2021_00517_99_t1ce/BraTS2021_00517_99_t1ce_t1.jpg]

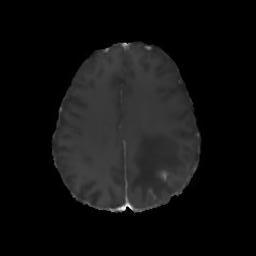

Supplement: Supplementary file 1 [file Data_Sheet_1.ZIP › Supplementary materials/evaluate/BraTS2021_00517_99_t1ce/BraTS2021_00517_99_t1ce_t1_flair.jpg]

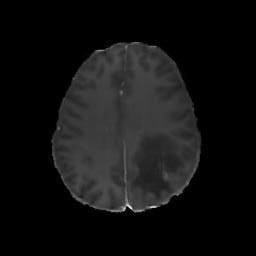

Supplement: Supplementary file 1 [file Data_Sheet_1.ZIP › Supplementary materials/evaluate/BraTS2021_00517_99_t1ce/BraTS2021_00517_99_t1ce_t1_t2.jpg]

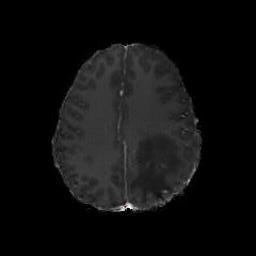

Supplement: Supplementary file 1 [file Data_Sheet_1.ZIP › Supplementary materials/evaluate/BraTS2021_00517_99_t1ce/BraTS2021_00517_99_t1ce_t2.jpg]

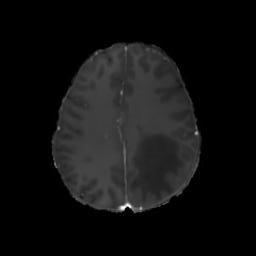

Supplement: Supplementary file 1 [file Data_Sheet_1.ZIP › Supplementary materials/evaluate/BraTS2021_00517_99_t1ce/BraTS2021_00517_99_t1ce_t2_flair.jpg]

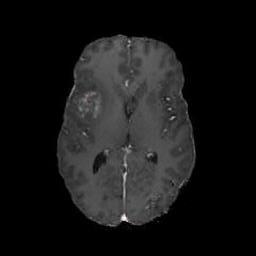

Supplement: Supplementary file 1 [file Data_Sheet_1.ZIP › Supplementary materials/evaluate/BraTS2021_00518_67_t1ce/BraTS2021_00518_67_t1ce_flair.jpg]

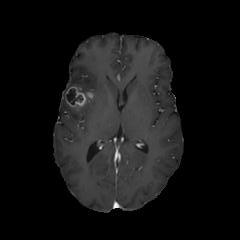

Supplement: Supplementary file 1 [file Data_Sheet_1.ZIP › Supplementary materials/evaluate/BraTS2021_00518_67_t1ce/BraTS2021_00518_67_t1ce_real.jpg]

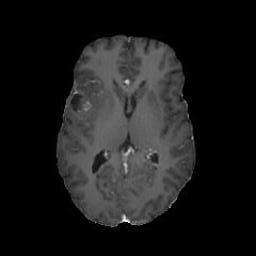

Supplement: Supplementary file 1 [file Data_Sheet_1.ZIP › Supplementary materials/evaluate/BraTS2021_00518_67_t1ce/BraTS2021_00518_67_t1ce_t1.jpg]

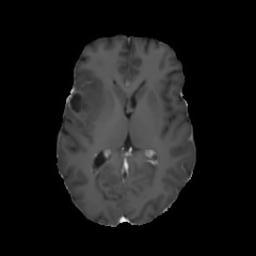

Supplement: Supplementary file 1 [file Data_Sheet_1.ZIP › Supplementary materials/evaluate/BraTS2021_00518_67_t1ce/BraTS2021_00518_67_t1ce_t1_flair.jpg]
