# Supplementary figures and images for: Multi-sequence generative adversarial network: better generation for enhanced magnetic resonance imaging images (part 1 of 2)
Source: Front Comput Neurosci. 2024 May 22;18:1365238. doi: 10.3389/fncom.2024.1365238 (PMC11151883; doi:10.3389/fncom.2024.1365238)

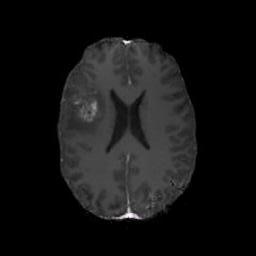

Supplement: Supplementary file 1 [file Data_Sheet_1.ZIP › Supplementary materials/evaluate/BraTS2021_00500_82_t1ce/BraTS2021_00500_82_t1ce_flair.jpg]

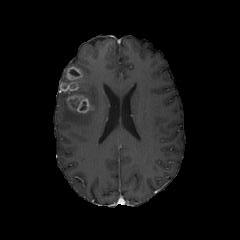

Supplement: Supplementary file 1 [file Data_Sheet_1.ZIP › Supplementary materials/evaluate/BraTS2021_00500_82_t1ce/BraTS2021_00500_82_t1ce_real.jpg]

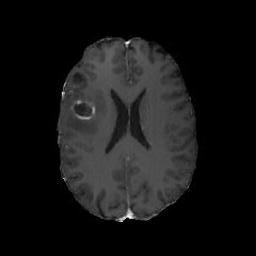

Supplement: Supplementary file 1 [file Data_Sheet_1.ZIP › Supplementary materials/evaluate/BraTS2021_00500_82_t1ce/BraTS2021_00500_82_t1ce_t1.jpg]

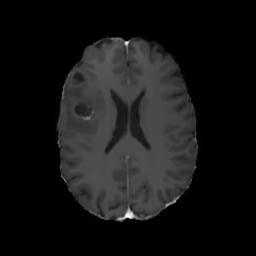

Supplement: Supplementary file 1 [file Data_Sheet_1.ZIP › Supplementary materials/evaluate/BraTS2021_00500_82_t1ce/BraTS2021_00500_82_t1ce_t1_flair.jpg]

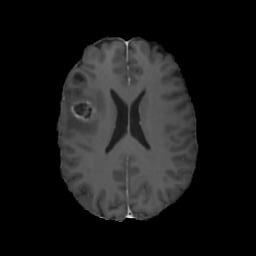

Supplement: Supplementary file 1 [file Data_Sheet_1.ZIP › Supplementary materials/evaluate/BraTS2021_00500_82_t1ce/BraTS2021_00500_82_t1ce_t1_t2.jpg]

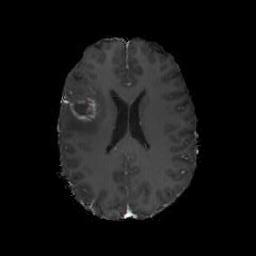

Supplement: Supplementary file 1 [file Data_Sheet_1.ZIP › Supplementary materials/evaluate/BraTS2021_00500_82_t1ce/BraTS2021_00500_82_t1ce_t2.jpg]

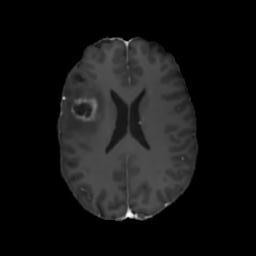

Supplement: Supplementary file 1 [file Data_Sheet_1.ZIP › Supplementary materials/evaluate/BraTS2021_00500_82_t1ce/BraTS2021_00500_82_t1ce_t2_flair.jpg]

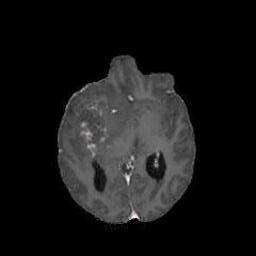

Supplement: Supplementary file 1 [file Data_Sheet_1.ZIP › Supplementary materials/evaluate/BraTS2021_00501_67_t1ce/BraTS2021_00501_67_t1ce_flair.jpg]

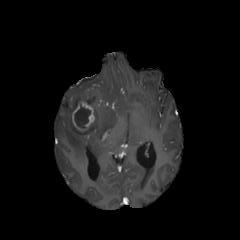

Supplement: Supplementary file 1 [file Data_Sheet_1.ZIP › Supplementary materials/evaluate/BraTS2021_00501_67_t1ce/BraTS2021_00501_67_t1ce_real.jpg]

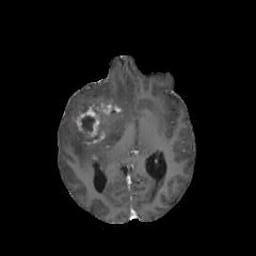

Supplement: Supplementary file 1 [file Data_Sheet_1.ZIP › Supplementary materials/evaluate/BraTS2021_00501_67_t1ce/BraTS2021_00501_67_t1ce_t1.jpg]

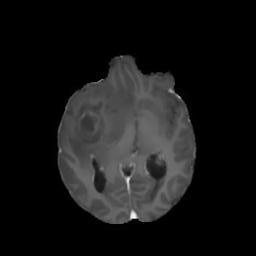

Supplement: Supplementary file 1 [file Data_Sheet_1.ZIP › Supplementary materials/evaluate/BraTS2021_00501_67_t1ce/BraTS2021_00501_67_t1ce_t1_flair.jpg]

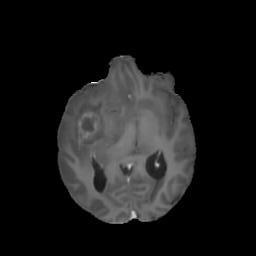

Supplement: Supplementary file 1 [file Data_Sheet_1.ZIP › Supplementary materials/evaluate/BraTS2021_00501_67_t1ce/BraTS2021_00501_67_t1ce_t1_t2.jpg]

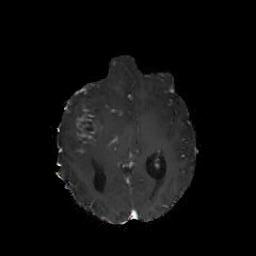

Supplement: Supplementary file 1 [file Data_Sheet_1.ZIP › Supplementary materials/evaluate/BraTS2021_00501_67_t1ce/BraTS2021_00501_67_t1ce_t2.jpg]

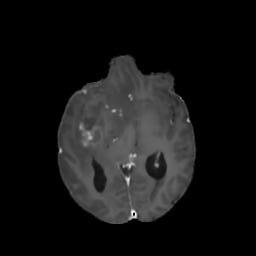

Supplement: Supplementary file 1 [file Data_Sheet_1.ZIP › Supplementary materials/evaluate/BraTS2021_00501_67_t1ce/BraTS2021_00501_67_t1ce_t2_flair.jpg]

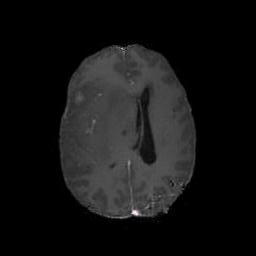

Supplement: Supplementary file 1 [file Data_Sheet_1.ZIP › Supplementary materials/evaluate/BraTS2021_00501_88_t1ce/BraTS2021_00501_88_t1ce_flair.jpg]

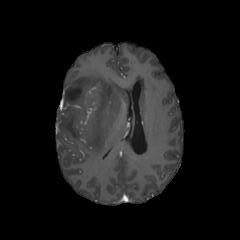

Supplement: Supplementary file 1 [file Data_Sheet_1.ZIP › Supplementary materials/evaluate/BraTS2021_00501_88_t1ce/BraTS2021_00501_88_t1ce_real.jpg]

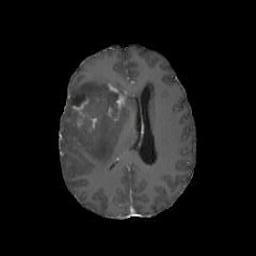

Supplement: Supplementary file 1 [file Data_Sheet_1.ZIP › Supplementary materials/evaluate/BraTS2021_00501_88_t1ce/BraTS2021_00501_88_t1ce_t1.jpg]

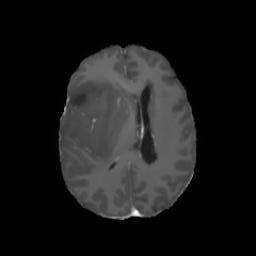

Supplement: Supplementary file 1 [file Data_Sheet_1.ZIP › Supplementary materials/evaluate/BraTS2021_00501_88_t1ce/BraTS2021_00501_88_t1ce_t1_flair.jpg]

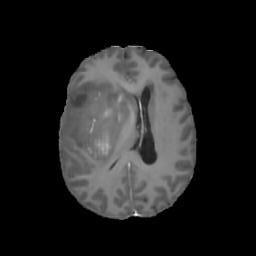

Supplement: Supplementary file 1 [file Data_Sheet_1.ZIP › Supplementary materials/evaluate/BraTS2021_00501_88_t1ce/BraTS2021_00501_88_t1ce_t1_t2.jpg]

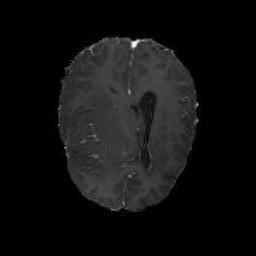

Supplement: Supplementary file 1 [file Data_Sheet_1.ZIP › Supplementary materials/evaluate/BraTS2021_00501_88_t1ce/BraTS2021_00501_88_t1ce_t2.jpg]

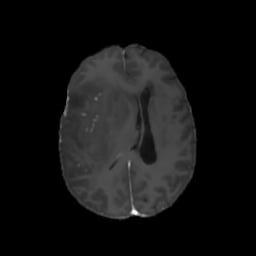

Supplement: Supplementary file 1 [file Data_Sheet_1.ZIP › Supplementary materials/evaluate/BraTS2021_00501_88_t1ce/BraTS2021_00501_88_t1ce_t2_flair.jpg]

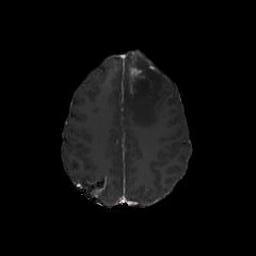

Supplement: Supplementary file 1 [file Data_Sheet_1.ZIP › Supplementary materials/evaluate/BraTS2021_00504_110_t1ce/BraTS2021_00504_110_t1ce_flair.jpg]

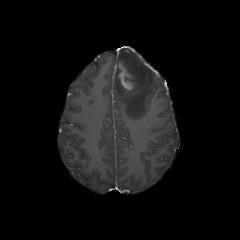

Supplement: Supplementary file 1 [file Data_Sheet_1.ZIP › Supplementary materials/evaluate/BraTS2021_00504_110_t1ce/BraTS2021_00504_110_t1ce_real.jpg]

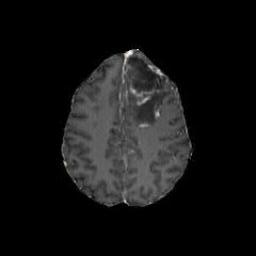

Supplement: Supplementary file 1 [file Data_Sheet_1.ZIP › Supplementary materials/evaluate/BraTS2021_00504_110_t1ce/BraTS2021_00504_110_t1ce_t1.jpg]

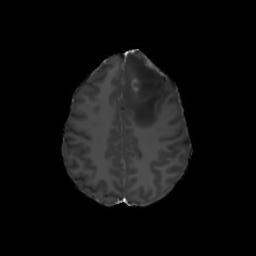

Supplement: Supplementary file 1 [file Data_Sheet_1.ZIP › Supplementary materials/evaluate/BraTS2021_00504_110_t1ce/BraTS2021_00504_110_t1ce_t1_flair.jpg]

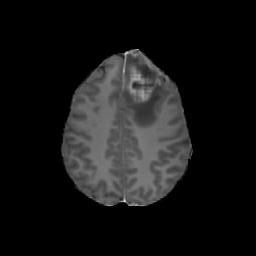

Supplement: Supplementary file 1 [file Data_Sheet_1.ZIP › Supplementary materials/evaluate/BraTS2021_00504_110_t1ce/BraTS2021_00504_110_t1ce_t1_t2.jpg]

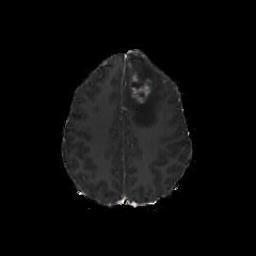

Supplement: Supplementary file 1 [file Data_Sheet_1.ZIP › Supplementary materials/evaluate/BraTS2021_00504_110_t1ce/BraTS2021_00504_110_t1ce_t2.jpg]

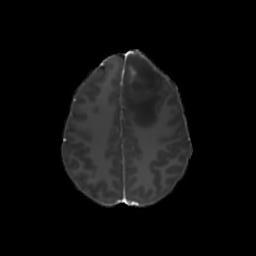

Supplement: Supplementary file 1 [file Data_Sheet_1.ZIP › Supplementary materials/evaluate/BraTS2021_00504_110_t1ce/BraTS2021_00504_110_t1ce_t2_flair.jpg]

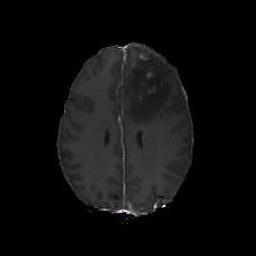

Supplement: Supplementary file 1 [file Data_Sheet_1.ZIP › Supplementary materials/evaluate/BraTS2021_00504_97_t1ce/BraTS2021_00504_97_t1ce_flair.jpg]

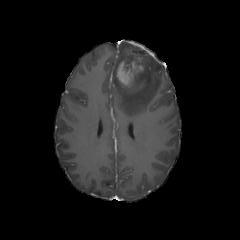

Supplement: Supplementary file 1 [file Data_Sheet_1.ZIP › Supplementary materials/evaluate/BraTS2021_00504_97_t1ce/BraTS2021_00504_97_t1ce_real.jpg]

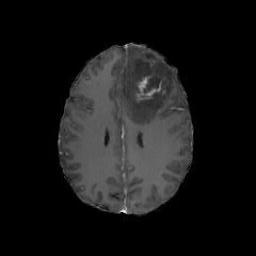

Supplement: Supplementary file 1 [file Data_Sheet_1.ZIP › Supplementary materials/evaluate/BraTS2021_00504_97_t1ce/BraTS2021_00504_97_t1ce_t1.jpg]

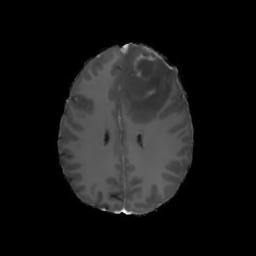

Supplement: Supplementary file 1 [file Data_Sheet_1.ZIP › Supplementary materials/evaluate/BraTS2021_00504_97_t1ce/BraTS2021_00504_97_t1ce_t1_flair.jpg]

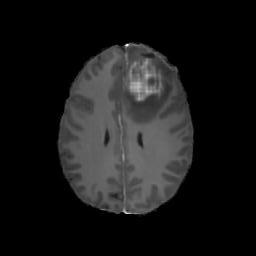

Supplement: Supplementary file 1 [file Data_Sheet_1.ZIP › Supplementary materials/evaluate/BraTS2021_00504_97_t1ce/BraTS2021_00504_97_t1ce_t1_t2.jpg]

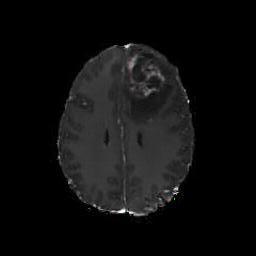

Supplement: Supplementary file 1 [file Data_Sheet_1.ZIP › Supplementary materials/evaluate/BraTS2021_00504_97_t1ce/BraTS2021_00504_97_t1ce_t2.jpg]

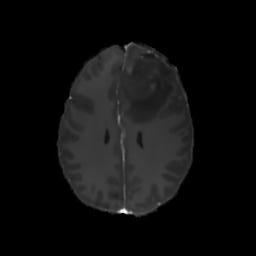

Supplement: Supplementary file 1 [file Data_Sheet_1.ZIP › Supplementary materials/evaluate/BraTS2021_00504_97_t1ce/BraTS2021_00504_97_t1ce_t2_flair.jpg]

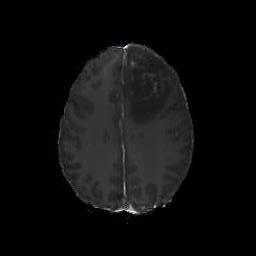

Supplement: Supplementary file 1 [file Data_Sheet_1.ZIP › Supplementary materials/evaluate/BraTS2021_00504_99_t1ce/BraTS2021_00504_99_t1ce_flair.jpg]

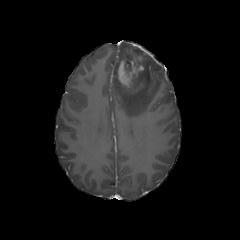

Supplement: Supplementary file 1 [file Data_Sheet_1.ZIP › Supplementary materials/evaluate/BraTS2021_00504_99_t1ce/BraTS2021_00504_99_t1ce_real.jpg]

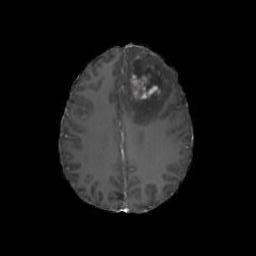

Supplement: Supplementary file 1 [file Data_Sheet_1.ZIP › Supplementary materials/evaluate/BraTS2021_00504_99_t1ce/BraTS2021_00504_99_t1ce_t1.jpg]

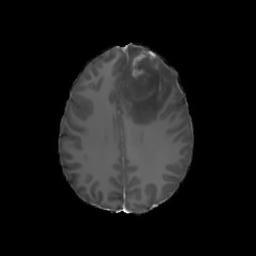

Supplement: Supplementary file 1 [file Data_Sheet_1.ZIP › Supplementary materials/evaluate/BraTS2021_00504_99_t1ce/BraTS2021_00504_99_t1ce_t1_flair.jpg]

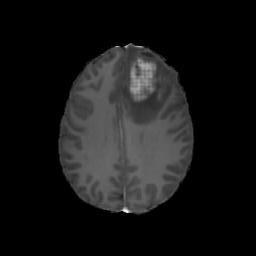

Supplement: Supplementary file 1 [file Data_Sheet_1.ZIP › Supplementary materials/evaluate/BraTS2021_00504_99_t1ce/BraTS2021_00504_99_t1ce_t1_t2.jpg]

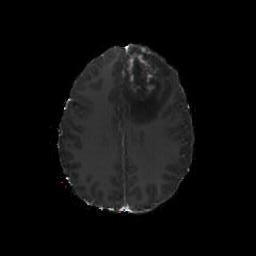

Supplement: Supplementary file 1 [file Data_Sheet_1.ZIP › Supplementary materials/evaluate/BraTS2021_00504_99_t1ce/BraTS2021_00504_99_t1ce_t2.jpg]

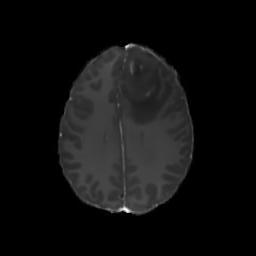

Supplement: Supplementary file 1 [file Data_Sheet_1.ZIP › Supplementary materials/evaluate/BraTS2021_00504_99_t1ce/BraTS2021_00504_99_t1ce_t2_flair.jpg]

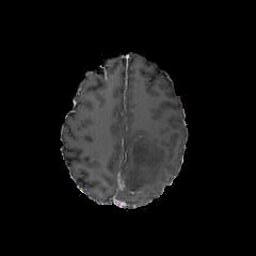

Supplement: Supplementary file 1 [file Data_Sheet_1.ZIP › Supplementary materials/evaluate/BraTS2021_00506_112_t1ce/BraTS2021_00506_112_t1ce_flair.jpg]

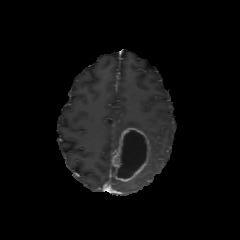

Supplement: Supplementary file 1 [file Data_Sheet_1.ZIP › Supplementary materials/evaluate/BraTS2021_00506_112_t1ce/BraTS2021_00506_112_t1ce_real.jpg]

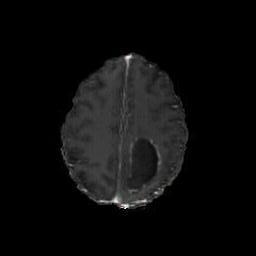

Supplement: Supplementary file 1 [file Data_Sheet_1.ZIP › Supplementary materials/evaluate/BraTS2021_00506_112_t1ce/BraTS2021_00506_112_t1ce_t1.jpg]

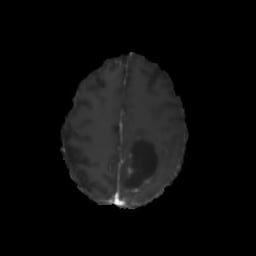

Supplement: Supplementary file 1 [file Data_Sheet_1.ZIP › Supplementary materials/evaluate/BraTS2021_00506_112_t1ce/BraTS2021_00506_112_t1ce_t1_flair.jpg]

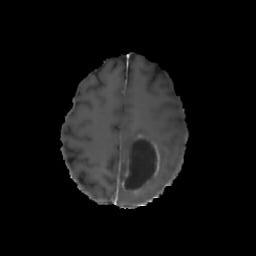

Supplement: Supplementary file 1 [file Data_Sheet_1.ZIP › Supplementary materials/evaluate/BraTS2021_00506_112_t1ce/BraTS2021_00506_112_t1ce_t1_t2.jpg]

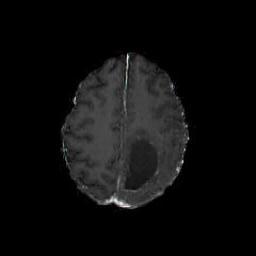

Supplement: Supplementary file 1 [file Data_Sheet_1.ZIP › Supplementary materials/evaluate/BraTS2021_00506_112_t1ce/BraTS2021_00506_112_t1ce_t2.jpg]

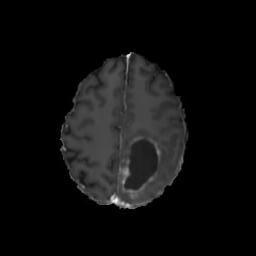

Supplement: Supplementary file 1 [file Data_Sheet_1.ZIP › Supplementary materials/evaluate/BraTS2021_00506_112_t1ce/BraTS2021_00506_112_t1ce_t2_flair.jpg]

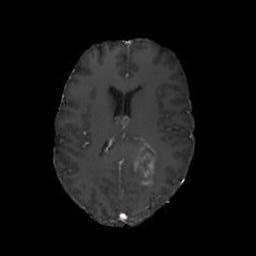

Supplement: Supplementary file 1 [file Data_Sheet_1.ZIP › Supplementary materials/evaluate/BraTS2021_00506_82_t1ce/BraTS2021_00506_82_t1ce_flair.jpg]

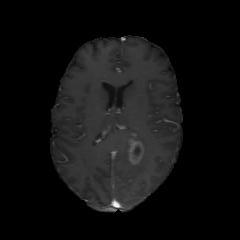

Supplement: Supplementary file 1 [file Data_Sheet_1.ZIP › Supplementary materials/evaluate/BraTS2021_00506_82_t1ce/BraTS2021_00506_82_t1ce_real.jpg]

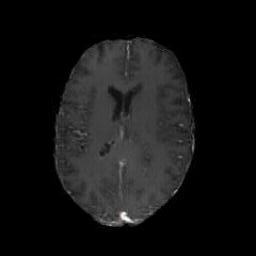

Supplement: Supplementary file 1 [file Data_Sheet_1.ZIP › Supplementary materials/evaluate/BraTS2021_00506_82_t1ce/BraTS2021_00506_82_t1ce_t1.jpg]

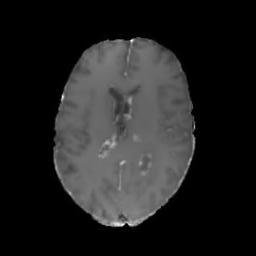

Supplement: Supplementary file 1 [file Data_Sheet_1.ZIP › Supplementary materials/evaluate/BraTS2021_00506_82_t1ce/BraTS2021_00506_82_t1ce_t1_flair.jpg]

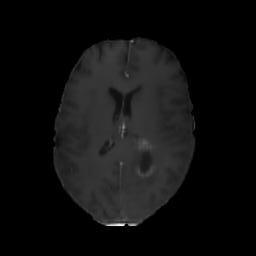

Supplement: Supplementary file 1 [file Data_Sheet_1.ZIP › Supplementary materials/evaluate/BraTS2021_00506_82_t1ce/BraTS2021_00506_82_t1ce_t1_t2.jpg]

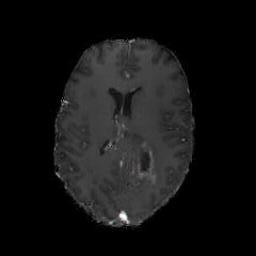

Supplement: Supplementary file 1 [file Data_Sheet_1.ZIP › Supplementary materials/evaluate/BraTS2021_00506_82_t1ce/BraTS2021_00506_82_t1ce_t2.jpg]

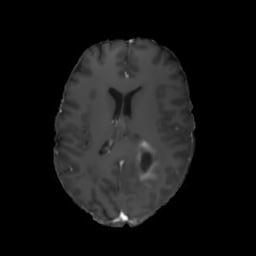

Supplement: Supplementary file 1 [file Data_Sheet_1.ZIP › Supplementary materials/evaluate/BraTS2021_00506_82_t1ce/BraTS2021_00506_82_t1ce_t2_flair.jpg]

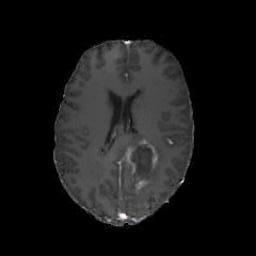

Supplement: Supplementary file 1 [file Data_Sheet_1.ZIP › Supplementary materials/evaluate/BraTS2021_00506_85_t1ce/BraTS2021_00506_85_t1ce_flair.jpg]

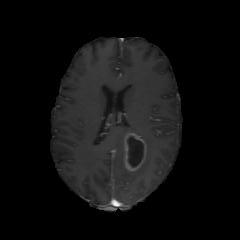

Supplement: Supplementary file 1 [file Data_Sheet_1.ZIP › Supplementary materials/evaluate/BraTS2021_00506_85_t1ce/BraTS2021_00506_85_t1ce_real.jpg]

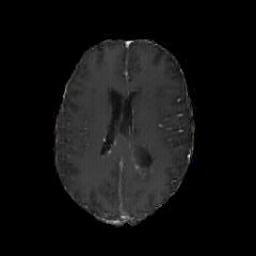

Supplement: Supplementary file 1 [file Data_Sheet_1.ZIP › Supplementary materials/evaluate/BraTS2021_00506_85_t1ce/BraTS2021_00506_85_t1ce_t1.jpg]

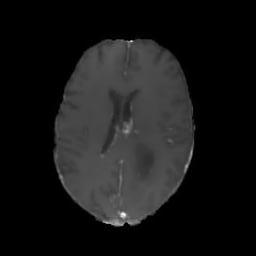

Supplement: Supplementary file 1 [file Data_Sheet_1.ZIP › Supplementary materials/evaluate/BraTS2021_00506_85_t1ce/BraTS2021_00506_85_t1ce_t1_flair.jpg]

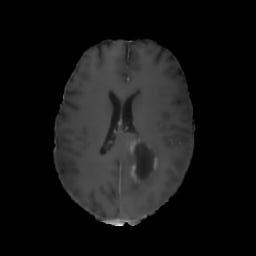

Supplement: Supplementary file 1 [file Data_Sheet_1.ZIP › Supplementary materials/evaluate/BraTS2021_00506_85_t1ce/BraTS2021_00506_85_t1ce_t1_t2.jpg]

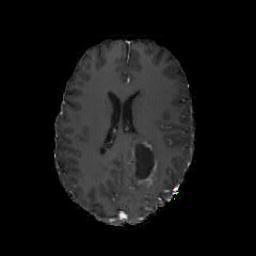

Supplement: Supplementary file 1 [file Data_Sheet_1.ZIP › Supplementary materials/evaluate/BraTS2021_00506_85_t1ce/BraTS2021_00506_85_t1ce_t2.jpg]

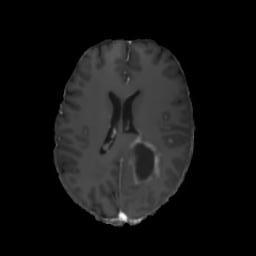

Supplement: Supplementary file 1 [file Data_Sheet_1.ZIP › Supplementary materials/evaluate/BraTS2021_00506_85_t1ce/BraTS2021_00506_85_t1ce_t2_flair.jpg]

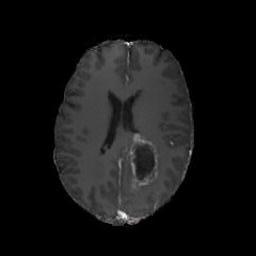

Supplement: Supplementary file 1 [file Data_Sheet_1.ZIP › Supplementary materials/evaluate/BraTS2021_00506_86_t1ce/BraTS2021_00506_86_t1ce_flair.jpg]

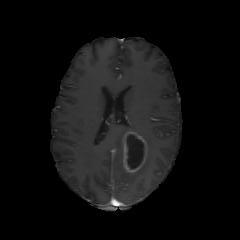

Supplement: Supplementary file 1 [file Data_Sheet_1.ZIP › Supplementary materials/evaluate/BraTS2021_00506_86_t1ce/BraTS2021_00506_86_t1ce_real.jpg]

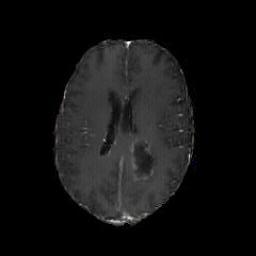

Supplement: Supplementary file 1 [file Data_Sheet_1.ZIP › Supplementary materials/evaluate/BraTS2021_00506_86_t1ce/BraTS2021_00506_86_t1ce_t1.jpg]

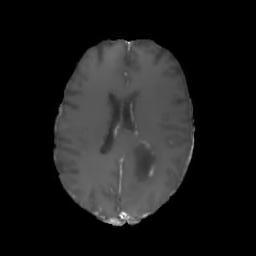

Supplement: Supplementary file 1 [file Data_Sheet_1.ZIP › Supplementary materials/evaluate/BraTS2021_00506_86_t1ce/BraTS2021_00506_86_t1ce_t1_flair.jpg]

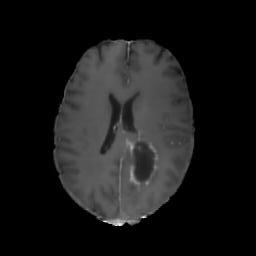

Supplement: Supplementary file 1 [file Data_Sheet_1.ZIP › Supplementary materials/evaluate/BraTS2021_00506_86_t1ce/BraTS2021_00506_86_t1ce_t1_t2.jpg]

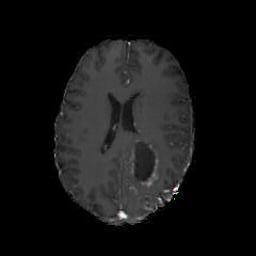

Supplement: Supplementary file 1 [file Data_Sheet_1.ZIP › Supplementary materials/evaluate/BraTS2021_00506_86_t1ce/BraTS2021_00506_86_t1ce_t2.jpg]

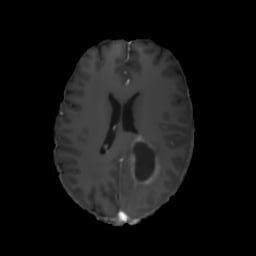

Supplement: Supplementary file 1 [file Data_Sheet_1.ZIP › Supplementary materials/evaluate/BraTS2021_00506_86_t1ce/BraTS2021_00506_86_t1ce_t2_flair.jpg]

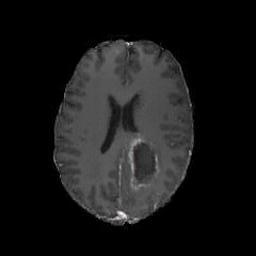

Supplement: Supplementary file 1 [file Data_Sheet_1.ZIP › Supplementary materials/evaluate/BraTS2021_00506_87_t1ce/BraTS2021_00506_87_t1ce_flair.jpg]

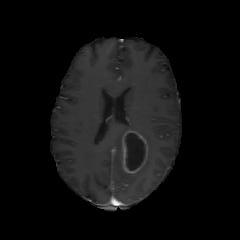

Supplement: Supplementary file 1 [file Data_Sheet_1.ZIP › Supplementary materials/evaluate/BraTS2021_00506_87_t1ce/BraTS2021_00506_87_t1ce_real.jpg]

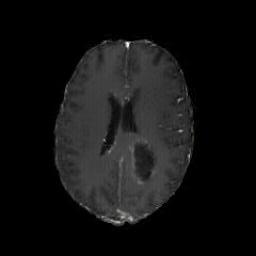

Supplement: Supplementary file 1 [file Data_Sheet_1.ZIP › Supplementary materials/evaluate/BraTS2021_00506_87_t1ce/BraTS2021_00506_87_t1ce_t1.jpg]

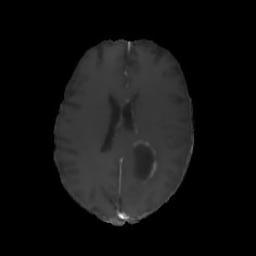

Supplement: Supplementary file 1 [file Data_Sheet_1.ZIP › Supplementary materials/evaluate/BraTS2021_00506_87_t1ce/BraTS2021_00506_87_t1ce_t1_flair.jpg]

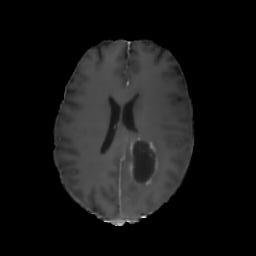

Supplement: Supplementary file 1 [file Data_Sheet_1.ZIP › Supplementary materials/evaluate/BraTS2021_00506_87_t1ce/BraTS2021_00506_87_t1ce_t1_t2.jpg]

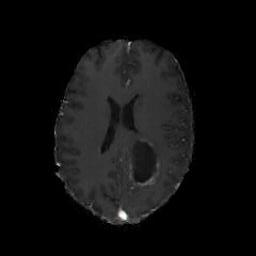

Supplement: Supplementary file 1 [file Data_Sheet_1.ZIP › Supplementary materials/evaluate/BraTS2021_00506_87_t1ce/BraTS2021_00506_87_t1ce_t2.jpg]

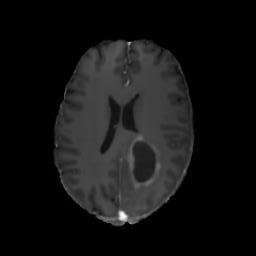

Supplement: Supplementary file 1 [file Data_Sheet_1.ZIP › Supplementary materials/evaluate/BraTS2021_00506_87_t1ce/BraTS2021_00506_87_t1ce_t2_flair.jpg]

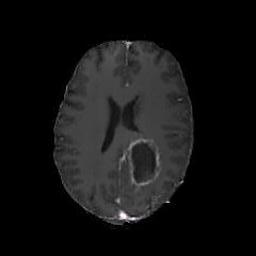

Supplement: Supplementary file 1 [file Data_Sheet_1.ZIP › Supplementary materials/evaluate/BraTS2021_00506_88_t1ce/BraTS2021_00506_88_t1ce_flair.jpg]

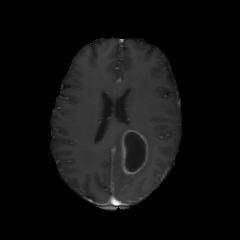

Supplement: Supplementary file 1 [file Data_Sheet_1.ZIP › Supplementary materials/evaluate/BraTS2021_00506_88_t1ce/BraTS2021_00506_88_t1ce_real.jpg]

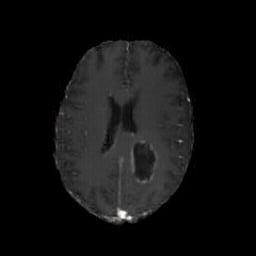

Supplement: Supplementary file 1 [file Data_Sheet_1.ZIP › Supplementary materials/evaluate/BraTS2021_00506_88_t1ce/BraTS2021_00506_88_t1ce_t1.jpg]

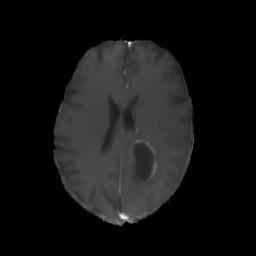

Supplement: Supplementary file 1 [file Data_Sheet_1.ZIP › Supplementary materials/evaluate/BraTS2021_00506_88_t1ce/BraTS2021_00506_88_t1ce_t1_flair.jpg]

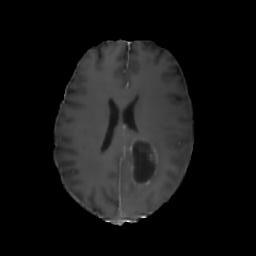

Supplement: Supplementary file 1 [file Data_Sheet_1.ZIP › Supplementary materials/evaluate/BraTS2021_00506_88_t1ce/BraTS2021_00506_88_t1ce_t1_t2.jpg]

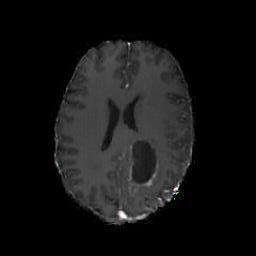

Supplement: Supplementary file 1 [file Data_Sheet_1.ZIP › Supplementary materials/evaluate/BraTS2021_00506_88_t1ce/BraTS2021_00506_88_t1ce_t2.jpg]

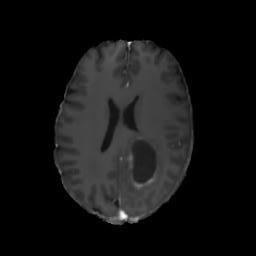

Supplement: Supplementary file 1 [file Data_Sheet_1.ZIP › Supplementary materials/evaluate/BraTS2021_00506_88_t1ce/BraTS2021_00506_88_t1ce_t2_flair.jpg]

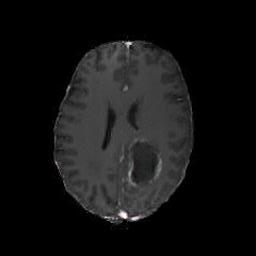

Supplement: Supplementary file 1 [file Data_Sheet_1.ZIP › Supplementary materials/evaluate/BraTS2021_00506_90_t1ce/BraTS2021_00506_90_t1ce_flair.jpg]

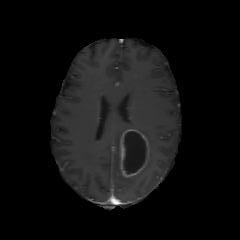

Supplement: Supplementary file 1 [file Data_Sheet_1.ZIP › Supplementary materials/evaluate/BraTS2021_00506_90_t1ce/BraTS2021_00506_90_t1ce_real.jpg]

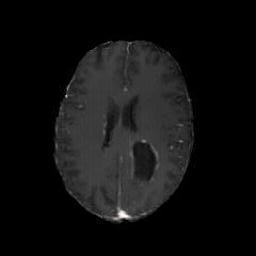

Supplement: Supplementary file 1 [file Data_Sheet_1.ZIP › Supplementary materials/evaluate/BraTS2021_00506_90_t1ce/BraTS2021_00506_90_t1ce_t1.jpg]

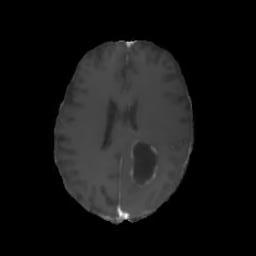

Supplement: Supplementary file 1 [file Data_Sheet_1.ZIP › Supplementary materials/evaluate/BraTS2021_00506_90_t1ce/BraTS2021_00506_90_t1ce_t1_flair.jpg]

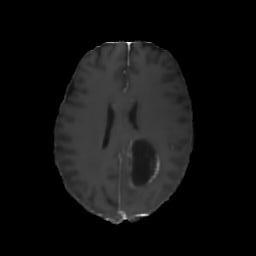

Supplement: Supplementary file 1 [file Data_Sheet_1.ZIP › Supplementary materials/evaluate/BraTS2021_00506_90_t1ce/BraTS2021_00506_90_t1ce_t1_t2.jpg]

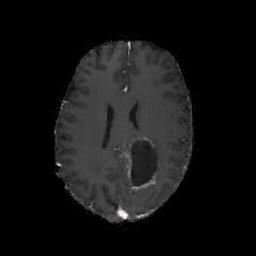

Supplement: Supplementary file 1 [file Data_Sheet_1.ZIP › Supplementary materials/evaluate/BraTS2021_00506_90_t1ce/BraTS2021_00506_90_t1ce_t2.jpg]

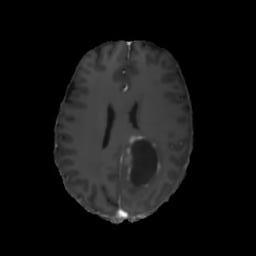

Supplement: Supplementary file 1 [file Data_Sheet_1.ZIP › Supplementary materials/evaluate/BraTS2021_00506_90_t1ce/BraTS2021_00506_90_t1ce_t2_flair.jpg]

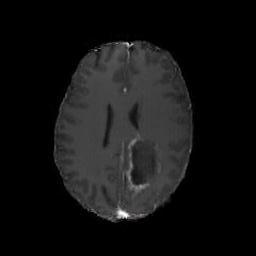

Supplement: Supplementary file 1 [file Data_Sheet_1.ZIP › Supplementary materials/evaluate/BraTS2021_00506_91_t1ce/BraTS2021_00506_91_t1ce_flair.jpg]

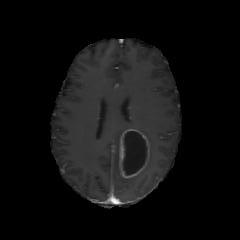

Supplement: Supplementary file 1 [file Data_Sheet_1.ZIP › Supplementary materials/evaluate/BraTS2021_00506_91_t1ce/BraTS2021_00506_91_t1ce_real.jpg]

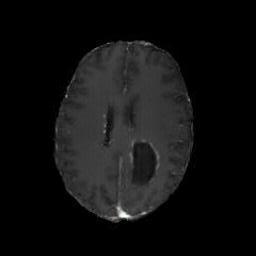

Supplement: Supplementary file 1 [file Data_Sheet_1.ZIP › Supplementary materials/evaluate/BraTS2021_00506_91_t1ce/BraTS2021_00506_91_t1ce_t1.jpg]

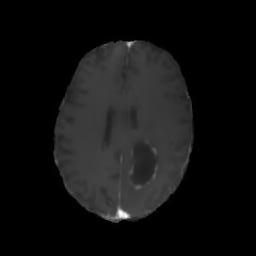

Supplement: Supplementary file 1 [file Data_Sheet_1.ZIP › Supplementary materials/evaluate/BraTS2021_00506_91_t1ce/BraTS2021_00506_91_t1ce_t1_flair.jpg]

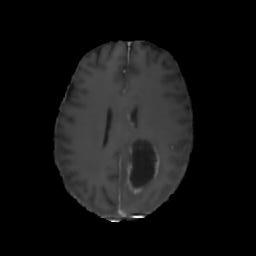

Supplement: Supplementary file 1 [file Data_Sheet_1.ZIP › Supplementary materials/evaluate/BraTS2021_00506_91_t1ce/BraTS2021_00506_91_t1ce_t1_t2.jpg]

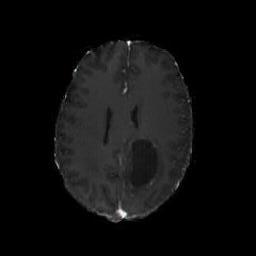

Supplement: Supplementary file 1 [file Data_Sheet_1.ZIP › Supplementary materials/evaluate/BraTS2021_00506_91_t1ce/BraTS2021_00506_91_t1ce_t2.jpg]

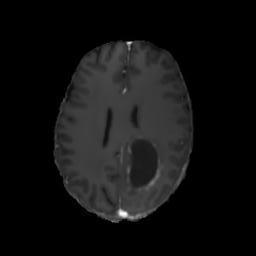

Supplement: Supplementary file 1 [file Data_Sheet_1.ZIP › Supplementary materials/evaluate/BraTS2021_00506_91_t1ce/BraTS2021_00506_91_t1ce_t2_flair.jpg]

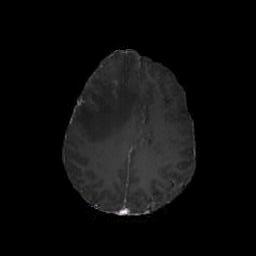

Supplement: Supplementary file 1 [file Data_Sheet_1.ZIP › Supplementary materials/evaluate/BraTS2021_00507_91_t1ce/BraTS2021_00507_91_t1ce_flair.jpg]

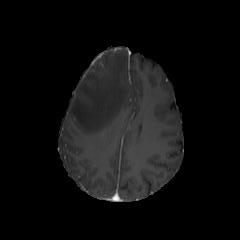

Supplement: Supplementary file 1 [file Data_Sheet_1.ZIP › Supplementary materials/evaluate/BraTS2021_00507_91_t1ce/BraTS2021_00507_91_t1ce_real.jpg]
